# AGC-YOLO: adaptive gated channel network for rice leaf disease detection

**DOI:** 10.3389/fpls.2026.1978719

**Published:** 2026-10-15

**Authors:** Fei Wang, Yuan Lv, Li Zhao, Jun Zhang, Yang Wen, Guo Li, Xianyao Zhang, Yuxiang Zhang

**Affiliations:** 1 Department of Statistics and Data Science, Southern University of Science and Technology, Shenzhen, China; 2 Department of Basic Education, Wuhu Vocational Technical University, Wuhu, China; 3 Anhui Longding Information Technology Co., Ltd, Wuhu, China; 4 School of Network Engineering, Wuhu Vocational Technical University, Wuhu, China; 5 School of Physics and Information Technology, Shaanxi Normal University, Xi’an, China

**Keywords:** adaptive gating, convolutional neural network, deep learning, rice disease detection, YOLO

## Abstract

Accurate detection of rice leaf diseases is critical for intelligent crop protection, yet class imbalance, degradation of multi-scale feature information, and the limited discriminative capability of feature fusion continue to hinder effective monitoring under field conditions. To address these challenges, this study presents AGC-YOLO, an improved detection model based on the YOLOv8s framework. First, a semantic-aware data augmentation network termed SAICP Net is devised to alleviate class imbalance and the scarcity of cross-class co-occurrence samples through a weakly supervised soft spatial prior and semantically constrained cross-category instance pasting. Second, a gated hybrid pooling module named GHP-SPPF is proposed to adaptively integrate max pooling and soft pooling via a learnable gating mechanism, thereby strengthening the discriminative representation of multi-scale lesions. Finally, a channel-adaptive fusion module termed CAFF is constructed to replace standard concatenation with channel-wise learnable weights and gated residual refinement, boosting the discriminative power of cross-scale feature fusion. Experimental results demonstrate that AGC-YOLO achieves an mAP@0.5 of 93.5 ± 0.6% on the rice disease dataset, representing a 3.0 percentage-point improvement over the baseline, and additional experiments on two public datasets further demonstrate the effectiveness of the proposed method for disease detection.

## 1 Introduction

Rice is one of the world’s most important food crops, underpinning the food security of more than half of the global population (Conde et al., 2025). However, rice production is continuously threatened by frequent outbreaks of leaf diseases—including rice blast, bacterial leaf blight, and brown spot—which cause substantial reductions in both yield and quality. Plant diseases cause global grain yield losses of up to 20–40% (Oerke, 2006). Ineffective disease management not only imposes substantial economic losses on farmers but also threatens the stability of food security systems. At present, rice leaf disease detection still relies heavily on manual visual inspection, which is labor-intensive, subjective, and dependent on specialized expertise; in resource-limited rural areas in particular, delayed diagnosis and misjudgment frequently lead to large-scale disease spread. Therefore, the development of intelligent, automated rice leaf disease detection techniques is essential for precise disease management and for safeguarding crop yield and farmer income.

Current crop disease detection methods can be broadly categorized into two paradigms: conventional machine learning (ML) and deep learning (DL). Conventional ML methods rely on handcrafted features; although they achieve competitive performance in small-scale, controlled environments, they exhibit notable limitations under complex field conditions. For instance, Khan et al. (2024) combined Gray Level Co-occurrence Matrix (GLCM) texture features with Scale-Invariant Feature Transform (SIFT) features for tomato disease recognition, achieving 92.3% accuracy with an SVM classifier. Nevertheless, their framework relies solely on texture information without incorporating complementary cues such as shape and color, and its performance is highly contingent on lesion segmentation accuracy, which restricts its generalization capability. Kainat et al. (2021) combined HOG, LBP, and color features for cucumber disease recognition, attaining an accuracy of 94.60%, yet handcrafted features remain poorly adaptable to complex field scenarios. Shrivastava et al. (Shrivastava and Pradhan, 2021) employed statistical color features for rice disease classification, achieving a maximum accuracy of 94.65%. They further noted that color features alone struggle to distinguish diseases with similar symptoms, revealing an inherent bottleneck in the representational capacity of handcrafted features. Overall, constrained by the limited representational power of handcrafted features, conventional methods cannot adequately address the challenges posed by pronounced inter-class morphological variation, substantial intra-class scale disparities in rice diseases, and complex field illumination conditions. Their robustness and generalization capability remain inadequate, limiting their applicability in practical agricultural production.

In contrast, deep learning enables end-to-end feature learning and autonomously extracts hierarchical abstract features, demonstrating significant advantages in handling complex backgrounds and emerging as the mainstream paradigm for intelligent crop disease detection. Based on their detection pipelines, existing deep learning-based disease detection methods can be broadly divided into two categories: two-stage and one-stage detectors. Two-stage methods adopt a cascade architecture in which region proposals are generated before classification and regression, and generally achieve higher detection accuracy. To address the large scale variation of rice lesions, Shi et al. (2026) integrated the SENet attention module and a multi-scale feature fusion module into Faster R-CNN, improving mAP by 14.9% over the baseline through loss function optimization. Alfred et al. (2025) developed a Mask R-CNN-based instance segmentation model for rice blast that enables pixel-level lesion detection and severity quantification. Nevertheless, the complex architectures of two-stage methods incur high computational overhead and a large parameter count, making them difficult to deploy on resource-constrained embedded or edge devices. This drawback limits their practical deployment in real-world agricultural applications.

One-stage methods, represented by the YOLO series, eliminate the need for separate region proposal generation and perform class prediction and bounding box regression directly and simultaneously on feature maps. By striking a favorable trade-off between inference speed and detection accuracy, they have become a prominent research focus in this field. In recent years, extensive improvements have been made to YOLO-based models tailored to the characteristics of rice diseases, with investigations spanning multiple dimensions, including lightweight design, feature enhancement, and cross-scenario generalization. For instance, Pan et al. (2025) proposed SSD-YOLO, which dynamically adjusts feature weights through the channel attention module (SENet) and optimizes the fusion of shallow and deep features via the dynamic upsampling module (DySample). While preserving the lightweight nature of the model, this approach significantly enhances the discriminability of disease regions against complex backgrounds, effectively reducing missed detections of dense disease objects under field conditions. Nevertheless, the model exhibits limited generalization to variations in field illumination and occlusion scenarios; furthermore, the adopted generic attention and upsampling modules are not specifically tailored to the pronounced scale variation across rice disease classes. From the perspective of feature interaction, Zhou et al. (Zhou and Wei, 2025) devised a Triplet Attention module for YOLO-DP. By decoupling channel, spatial, and positional attention, the module enables multi-dimensional feature correlation modeling and is further combined with wavelet convolution (WTConv) to strengthen the extraction of local texture information, enabling the model to maintain high detection accuracy even in scenarios with blurred lesion edges or missing texture details. However, this method is constrained by its reliance on large-scale, high-quality annotated data and its insufficient coverage of extreme scenarios; in addition, the individual contributions of its multiple improved modules have not been rigorously disentangled through component-wise ablation studies. Nguyen et al. (2026) focused on fine-grained deep feature extraction. They enhanced multi-scale contextual information by designing the ImDS-C3k2 feature pyramid module and introduced a Rice-IoU loss function tailored to rice disease characteristics to optimize bounding box regression for small objects. Consequently, the model achieves an mAP@0.5 of 56.1% on scenarios with tiny and dense disease lesions. Nonetheless, its overall detection accuracy remains relatively low, and the generalizability of the proposed modules has not been sufficiently validated; moreover, no targeted design addresses the severe inter-class scale variation and class imbalance in rice diseases. In addition, Cao et al. (2025) proposed GAF-Net, which substantially improves cross-scale detection performance for maize diseases through a global attention fusion module and an adaptive feature calibration layer. However, the model is validated only on maize as a single crop, and its cross-crop generalization performance remains unassessed; furthermore, the FASFF module requires uniform channel compression across three scales prior to feature fusion, which may cause detail loss for small lesions. Wang et al. (2022) designed MGA-YOLO, which integrates multi-granularity feature aggregation with an attention-guided decoder to achieve robust detection of apple diseases under complex illumination conditions. Collectively, these studies have advanced one-stage methods in terms of accuracy, efficiency, and generalizability through diverse technical routes, offering transferable paradigms not only for rice disease detection but also for intelligent monitoring of other crops.

The representative plant disease detection studies reviewed above are compared in **Table 1**. In summary, existing improved YOLO-based rice disease detection studies predominantly focus on the plug-and-play replacement of generic modules, while the combined challenges of class imbalance, substantial inter-class scale variation, feature degradation of dense small targets, and limited adaptability in cross-scale feature fusion remain insufficiently addressed. To address these limitations, this study proposes AGC-YOLO, a detection model incorporating three targeted modules—SAICP Net, GHP-SPPF, and CAFF—designed at three levels: data augmentation, pooling optimization, and fusion strategy. The primary contributions of this study are summarized as follows:

**Table 1:** Comparison of relevant literature in plant disease detection.

Category	Author	Method	Advantages	Disadvantages
Conventional Machine Learning Methods	Khan et al. (2024)	SVM;GLCM;SIFT Features	92.3% recognition accuracy	Only texture features; relies on lesion segmentation; poor generalization
	Kainat et al. (2021)	HOG;LBP; Color Features	94.6% recognition accuracy	Handcrafted features adapt poorly to complex field scenes
	Shrivastava et al (Shrivastava and Pradhan, 2021)	Statistical Color Features	94.7% classification accuracy	Hard to distinguish similar symptoms; weak feature representation
Two-stage Deep Learning Methods	Shi et al. (2026)	Faster R-CNN; SENet; Multi-scale Fusion	14.9% mAP improvement over baseline	High computational cost; hard to deploy on edge devices
	Alfred et al. (2025)	Mask R-CNN;Instance Segmentation	Pixel-level detection & severity quantification	Heavy network; low practical deploy ability
One-stage Deep Learning Methods	Pan et al. (2025)	YOLOv8;SENet; DySample	Lightweight; reduces dense lesion misdetection	Weak robustness to illumination and occlusion
	Zhou et al (Zhou and Wei, 2025)	YOLOv8;Triplet Attention;GLSA; WTConv; EIOU	High accuracy on blurred/texture-missing lesions	Requires large annotations; no ablation verification
	Nguyen et al. (2026)	YOLOv13; ImDS-C3k2;Rice-IoU	56.1% mAP@0.5 for tiny dense lesions	Low overall accuracy; no design for scale/class imbalance
	Cao et al. (2025)	YOLOv8;GSConv	Strong cross-scale detection performance	Only validated on maize; small lesion detail loss
	Wang et al. (2022)	YOLOv5;ALDOD Dataset; Ghost;CBAM	Robust under complex illumination	Only verified on apple; unvalidated for rice diseases

1. To address class imbalance and the scarcity of cross-class co-occurrence samples in the original dataset, SAICP Net is proposed to learn a soft lesion-associated plant-foreground prior in a weakly supervised manner and use it together with an IoU constraint to guide semantically plausible instance placement, rather than unconstrained random pasting.
2. The GHP-SPPF gated hybrid pooling module is designed to generate input-dependent channel-wise gates to adaptively fuse MaxPool and SoftPool features, thereby alleviating feature degradation for dense small targets while preserving complementary contextual information for lesions of different scales.
3. The CAFF channel-adaptive fusion module is constructed to replace standard static concatenation with learnable channel-wise weighting before feature fusion, followed by gated residual refinement, improving the quality of multi-scale feature interaction.
4. The effectiveness of each module is verified through ablation and comparative experiments on a public rice disease dataset, where AGC-YOLO achieves an mAP@0.5 of 93.5 ± 0.6%, a 3.0 percentage-point improvement over the YOLOv8s baseline. The performance of the proposed method is further evaluated on two additional public datasets.

The remainder of this paper is organized as follows. Section 2 describes the overall architecture of AGC-YOLO and its three key components—the SAICP Net augmentation strategy, the GHP-SPPF pooling module, and the CAFF fusion module. Section 3 presents the experimental settings, datasets, and evaluation metrics, followed by the comparative experiments, ablation studies, qualitative visualization and cross-dataset generalization analyses, together with a discussion of the results and the limitations of the present study. Section 4 concludes this paper and outlines directions for future work.

## 2 Materials and methods

### 2.1 Overall architecture

This section presents AGC-YOLO, an improved detection model based on YOLOv8s and specifically tailored for rice disease detection. The model inherits the CSP backbone and PAN-FPN neck of YOLOv8s and incorporates three complementary modules at three key positions—the data preprocessing pipeline, the end of the backbone, and the fusion nodes of the neck—to address three bottlenecks: data augmentation for long-tailed samples, information loss in multi-scale pooling, and performance degradation in cross-scale feature fusion. First, a learnable Semantic-Aware Instance Copy-Paste Network (SAICP Net) is introduced in the data preprocessing stage. By leveraging weakly supervised soft spatial prior to constrain the placement of pasted instances, it enables semantically plausible cross-class instance synthesis, thereby mitigating minority-class scarcity and the lack of cross-class co-occurrence samples under long-tailed distributions. Second, a Gated Hybrid Pooling module (GHP-SPPF) is embedded at the end of the backbone. It dynamically blends max pooling and soft pooling via channel-wise learnable gating, achieving an adaptive balance between preserving fine-grained details of tiny lesions and aggregating boundary context for large lesions. Finally, the Channel-Adaptive Feature Fusion (CAFF) module replaces all concatenation (Concat) operations in the PAN-FPN neck. It enables learnable channel-wise weighted fusion of deep and shallow features through channel-wise learnable weights and gated residual refinement. The overall architecture of AGC-YOLO is illustrated in **Figure 1**.

**Figure 1:**
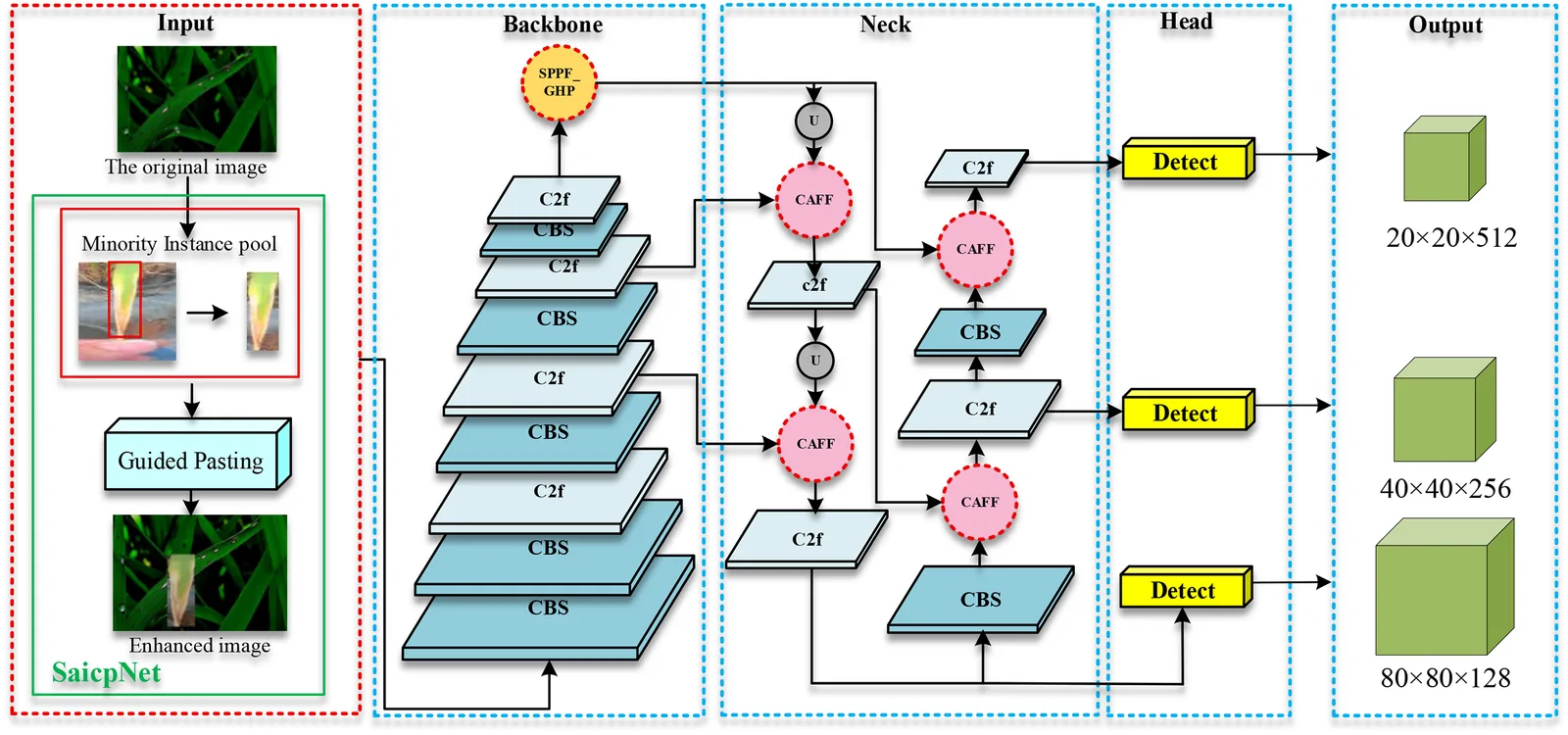
Network architecture of AGC-YOLO.

Rather than acting as three independent plug-and-play modules, these components form a cascaded optimization pipeline spanning data processing, feature extraction, and feature fusion. SAICP Net first expands the semantic coverage of training samples at the data source, enabling subsequent modules to learn from a more diverse data distribution. The augmented samples are then passed through the backbone, where GHP-SPPF preserves the sensitivity of max pooling to local extrema for tiny lesions while incorporating the exponential weighting of soft pooling to maintain boundary gradient integrity for large lesions. In the neck, CAFF independently recalibrates each input branch before fusion, preventing shallow details from being overshadowed by deep semantic features, while a learnable gated residual refinement provides channel-wise compensation for the fused features. This progressive design integrates data augmentation, feature preservation, and fusion optimization into a unified framework, enabling the modules to jointly improve detection accuracy without substantial parameter overhead.

### 2.2 SAICP Net

Instance-level augmentation methods such as Copy-Paste (Ghiasi et al., 2021) and CutMix (Yun et al., 2019) enhance sample diversity by cropping and pasting object regions, while subsequent studies have addressed class imbalance through probability sampling (Yang and Qiu, 2024) and diffusion-based synthesis (Qin et al., 2025). However, conventional instance-pasting strategies generally lack explicit semantic constraints on candidate placement, which may result in lesion instances being pasted onto visually implausible background regions. To address semantic placement inconsistency and class imbalance in instance copy-paste augmentation for rice disease detection (Wang et al., 2020b), this study presents SAICP Net, a semantic-aware instance copy-paste augmentation framework. By introducing a lightweight encoder-decoder-derived soft spatial prior into candidate placement screening, SAICP Net reduces semantically implausible instance placement and improves the diversity of minority-class training samples.

First, a lightweight encoder-decoder network, termed LeafSegNet, is introduced to generate a spatial probability map for guiding instance placement. The encoder consists of three stacked downsampling blocks, each comprising a convolutional layer with stride 2, a batch normalization (BN) layer, and a ReLU activation. The decoder consists of three cascaded upsampling blocks, each consisting of a nearest-neighbor upsampling layer, a convolutional layer with stride 1, a BN layer, and a ReLU activation. The output layer adopts a Sigmoid activation function in place of ReLU, producing a single-channel probability map *M*. Rather than representing a pixel-accurate leaf segmentation mask, *M* is interpreted as a soft lesion-associated plant-foreground prior learned from weak box-level supervision. The architecture of LeafSegNet is illustrated in **Figure 2**.

**Figure 2:**
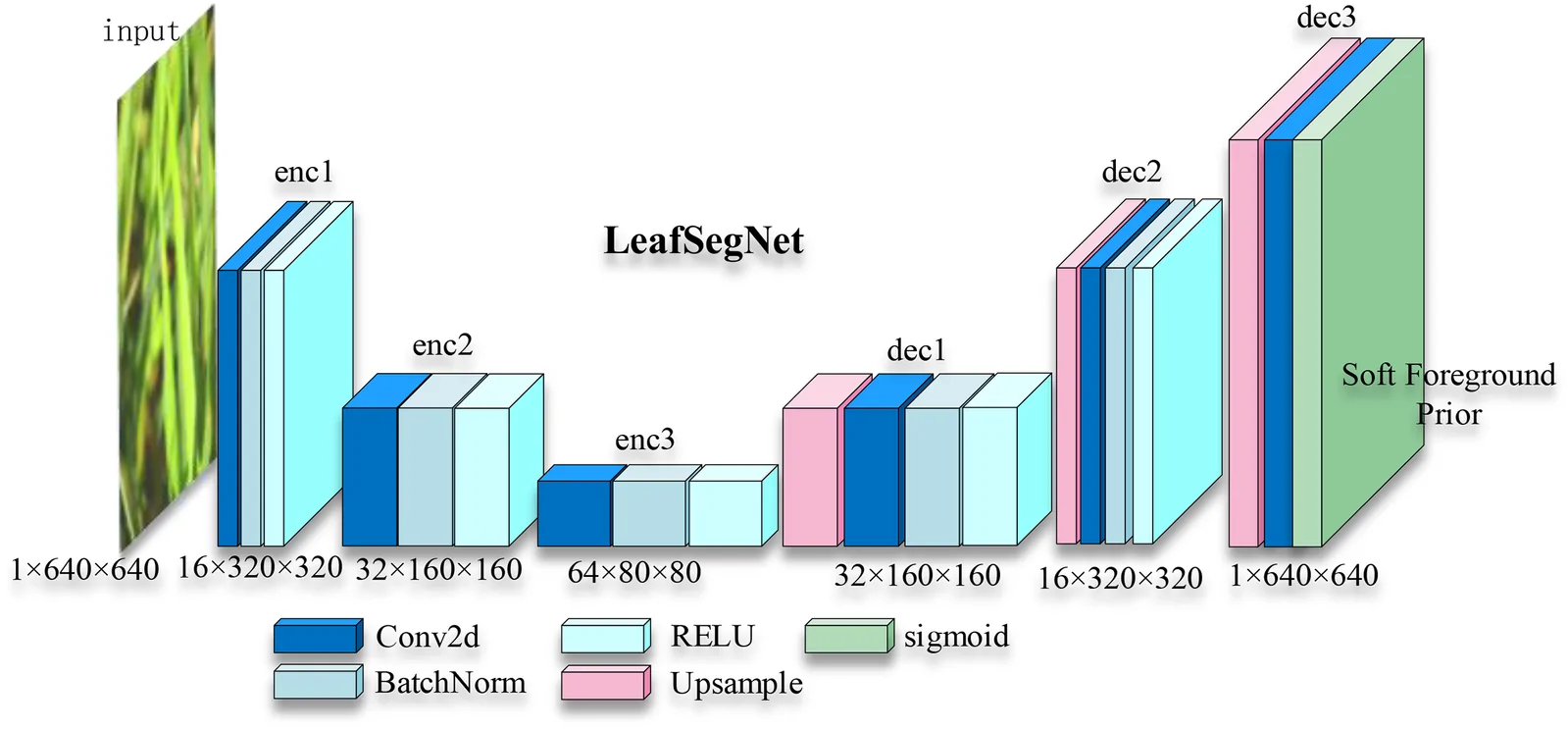
Network architecture of LeafSegNet.

LeafSegNet is optimized using binary cross-entropy (BCE) loss. Because the available annotations correspond to disease-lesion bounding boxes rather than complete leaf regions, the bounding boxes are used only as weak spatial supervision. For each training image, pixels located inside the ground-truth lesion bounding boxes are assigned positive labels, while pixels outside the boxes are assigned negative labels. The loss function is defined as Equation 2-1:

(2-1) $$L=-\frac{1}{HW}\sum_{i=1}^{HW}\left[y_{i}\log M_{i}+\left(1-y_{i}\right)\log\left(1-M_{i}\right)\right]$$

Where ${\tilde{M}}_{i}$ denotes the network’s prediction at pixel *i*, and *y_i_* is the corresponding weak supervision label. Since the supervision is derived from lesion-level bounding boxes, the resulting probability map is not regarded as an accurate segmentation of complete leaves. Instead, it serves as a soft spatial prior that tends to produce stronger responses in lesion-associated plant regions and lower responses in homogeneous background regions. During augmentation, this probability map is used directly without binarization and is combined with the IoU-based constraint to screen candidate paste positions. After obtaining the spatial probability map, SAICP Net performs minority-class instance synthesis. Let $\text{S}=\left\{{\text{S}}_{1},{\text{S}}_{2},\ldots ,{\text{S}}_{\text{k}}\right\}$ denote the source instance pool, which consists of annotated lesion instances of minority classes from the training set. Each element S_k_ corresponds to a lesion Region of Interest (ROI) cropped from original training images based on ground-truth bounding boxes. For each target training image *I*, the augmentation procedure is activated with probability *ρ* = 0.7. Upon activation n∈{1,2,3} source instances are randomly sampled from *S* via a sampling strategy that prioritizes minority classes. Let the selected source instance *S*_k_ have original dimensions w_s_×h_s_. Aspect-ratio-preserving random scaling is then applied to the instance as shown as in Equation 2-2:

(2-2) $${\{}_{h_{s}^{\prime }=\lambda \cdot h_{s}}^{w_{s}^{\prime }=\lambda \cdot w_{s}}\lambda ∼U\left(S_{\min},S_{\max}\right)$$

where the scaling factor λ is uniformly sampled from:


$$\left[S_{min},S_{max}\right]=\left[0.6,1.4\right]$$

This random scaling increases the scale diversity of synthesized lesion instances while avoiding excessive geometric distortion. The actual implementation uses one common scaling interval rather than class-specific scaling ranges.

For the scaled instance ${\text{S}}_{\text{k}}^{\prime }$, a candidate paste anchor point (x_c_, y_c_) is randomly sampled on the target image, yielding the candidate paste region $\text{R}=\left[{\text{x}}_{\text{c}}-\frac{{\text{w}}_{\text{s}}^{\prime }}{2},{\text{y}}_{\text{c}}-\frac{{\text{h}}_{\text{s}}^{\prime }}{2},{\text{x}}_{\text{c}}+\frac{{\text{w}}_{\text{s}}^{\prime }}{2},{\text{y}}_{\text{c}}+\frac{{\text{h}}_{\text{s}}^{\prime }}{2}\right]$. Two spatial constraints are jointly applied to determine whether the candidate location is accepted. First, the semantic plausibility of the candidate region r is evaluated using the mean response of the probability map as shown in Equation 2-3:

(2-3) $$r=\frac{1}{w_{s}^{\prime }\cdot h_{s}^{\prime }}\sum_{\left(x,y\right)\in R}M\left(x,y\right)\geq T_{1}$$

Where *M*(*x*, *y*) denotes the response at spatial position (*x*, *y*), and *T*_1_ is the foreground-response threshold. In this study, $T_{1}=0.15$. A candidate location satisfying this criterion is more likely to lie within lesion-associated plant regions rather than homogeneous background regions. Because the probability map is used as a permissive spatial prior rather than a strict region classifier, this constraint biases the paste position toward semantically plausible regions without requiring pixel-level segmentation. In the implementation, the mean value of the probability map within the candidate region is directly calculated without binarization.

Second, an occlusion-avoidance constraint is applied to prevent severe overlap between newly pasted instances and existing annotations. For a candidate region r, the IoU between r and each existing ground-truth bounding box must not exceed as given in Equation 2-4:

(2-4) $$T_{IOU}=0.03$$

If either the soft-foreground constraint or the IoU constraint is violated, another candidate location is randomly sampled. For each source instance, at most 30 candidate positions are attempted. If no valid position is found after the maximum number of attempts, the paste operation for the current source instance is discarded. The implementation applies the same two-stage screening process using the mean foreground response and the IoU threshold.

Once a valid paste position is determined, boundary feathering and a high-opacity blending are applied to reduce visible discontinuities between the pasted instance and the target image. Let $FЄ{\left[0,1\right]}^{h_{s}^{\prime }\times w_{s}^{\prime }}$ denote the two-dimensional feathering weight at position (*x*, *y*), where the weight remains high in the central region and gradually decreases toward the ROI boundaries is formulated in Equation 2-5:

(2-5) $$I^{\prime }\left(x,y\right)=\alpha \cdot F\left(x,y\right)\cdot S^{\prime }\left(x,y\right)+\left(1-\alpha \cdot F\left(x,y\right)\right)\cdot I\left(x,y\right)$$

where $S_{\left(x,y\right)}^{\prime }$ represents the pasted source instance, *I*(*x*, *y*) represents the original target image, and *α* controls the base opacity of the pasted instance. In the experiments, the feathering width is set to *δ* = 5, while *α* is randomly sampled from [0.85,1.0]. This smooth transition reduces hard-edge artifacts around the pasted region and improves the visual consistency of synthesized samples. These values correspond directly to the implemented feathering and alpha-blending procedure.

SAICP Net augmentation is applied only to the training subset, while the original validation and test subsets remain unchanged, thereby preventing augmented samples from entering the evaluation data. After SAICP Net augmentation, the number of training images increases from 934 to 1,370. The complete augmentation procedure is illustrated in **Figure 3**.

**Figure 3:**
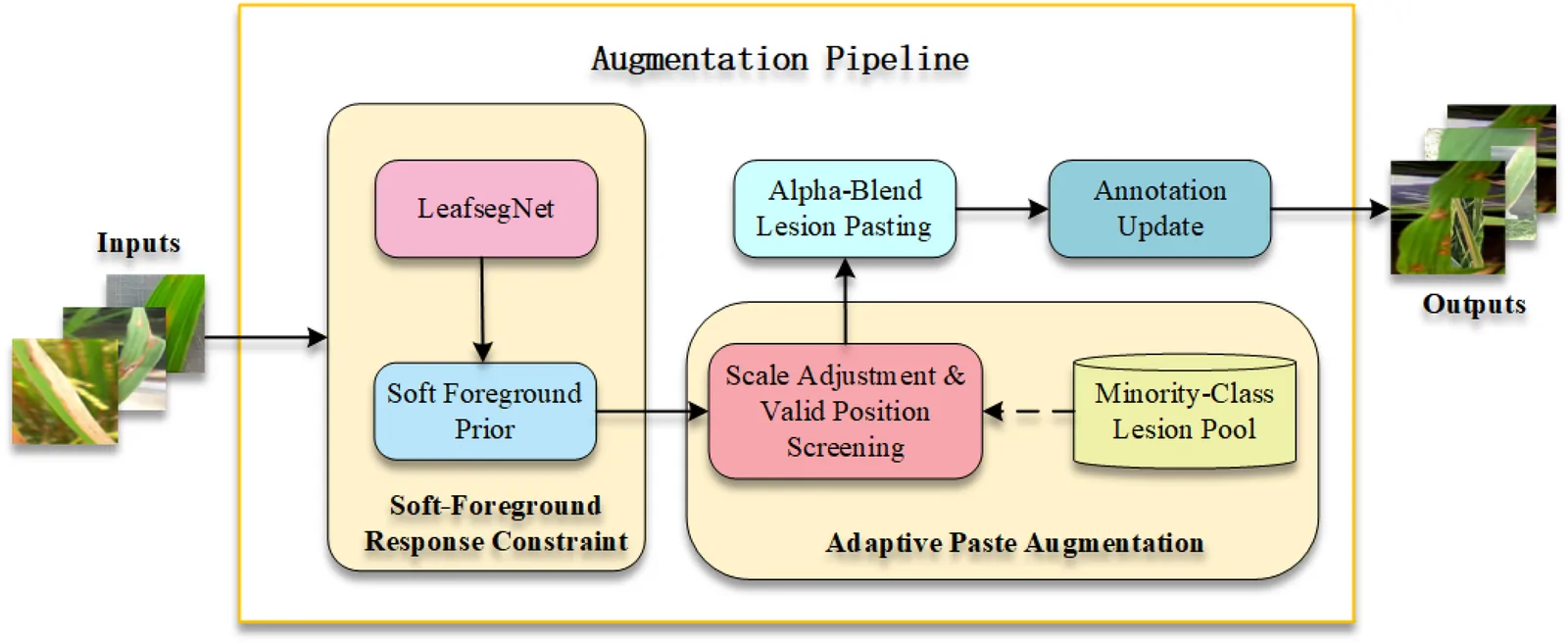
Complete data augmentation flowchart.

### 2.3 Gated hybrid pooling module

The SPPF module in YOLOv8 aggregates multi-scale features through cascaded max pooling, yet its hard selection discards weak activations from dense small targets. Recent studies have addressed this through soft pooling (Stergiou et al., 2021), adaptive weight blending (Guang et al., 2025), attention-integrated SPPF variants (Guo and Ren, 2025), and multi-branch pooling fusion (Fan et al., 2025), while SENet (Hu et al., 2018) and ECA-Net (Wang et al., 2020) provide *post-hoc* channel recalibration. However, these methods adopt scalar or globally shared blending weights, neglecting that optimal pooling strategies vary substantially across channels.

Rice diseases exhibit pronounced scale and morphological variations under field conditions. For instance, early-stage rice blast typically manifests as tiny punctate lesions with sharp boundaries, whereas bacterial leaf blight presents as large, diffuse lesions with blurred margins. Standard max pooling (MaxPool) uses a hard selection mechanism that retains only the maximum activation. Although it effectively captures high-frequency details of small lesions, it tends to lose boundary context for large lesions. By contrast, soft pooling (SoftPool) smooths feature responses via exponential weighting to preserve boundary integrity of large lesions, but reduces the saliency of small lesions due to weighted averaging.

To address these limitations, this study proposes a Gated Hybrid Pooling (GHP) module. As shown in **Figure 4**, for an input feature map $\text{X}\in {\text{R}}^{C\times H\times W}$ (where C is the number of input channels and H, W are the spatial dimensions), GHP runs MaxPool and SoftPool branches in parallel within a local sliding window (receptive field Ω), extracts complementary local features, and adaptively fuses them along the channel dimension through a data-driven channel-wise gating mechanism (Chavan et al., 2026).

**Figure 4:**
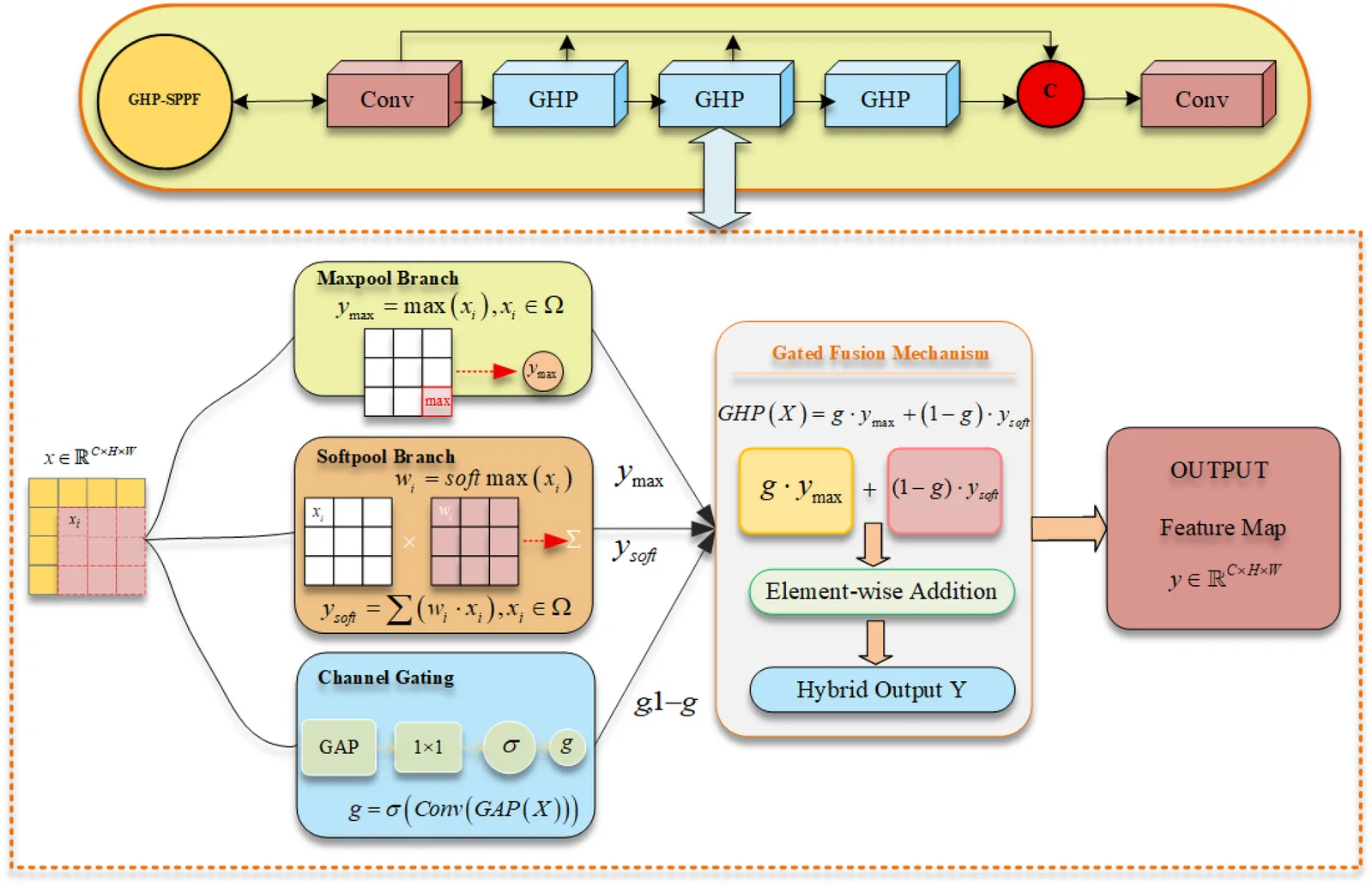
Architecture of the gated hybrid pooling module.

Specifically, for an arbitrary spatial location, a k×k local receptive field Ω is first defined. The set of feature values within this region is denoted as ${\left\{{\text{x}}_{\text{i}}\right\}}_{\text{i}=1}^{{\text{k}}^{2}}$, which comprises a total of k^2^ feature elements. Accordingly, the MaxPool and SoftPool branches adopt distinct strategies respectively:

The MaxPool branch yields the maximum activation within the receptive field as shown in Equation 2-6:

(2-6) $${\text{y}}_{\text{max}}=\text{max}\left({\text{x}}_{\text{i}}\right),{\text{x}}_{\text{i}}\in \text{Ω}$$

In the SoftPool branch, the normalized attention weight *w_i_* for each feature element *x_i_* within the receptive field Ω is first computed via the softmax function as computed in Equation 2-7:

(2-7) $$w_{i}=soft\max\left(x_{i}\right)=\frac{\exp\left(x_{i}\right)}{\sum_{j\in \Omega }\exp\left(x_{j}\right)}$$

As shown in **Figure 5**, exponential mapping amplifies linear differences in raw activation values into exponential-scale disparities. This design allows larger activation values to dominate the weight distribution, while smaller activation values are significantly attenuated rather than completely zeroed out. The resulting weight distribution combines selectivity with inclusiveness: it overcomes the hard-selection limitation of MaxPool, which discards all non-maximum signals, while suppressing interference from noisy activations in the aggregation output.

**Figure 5:**
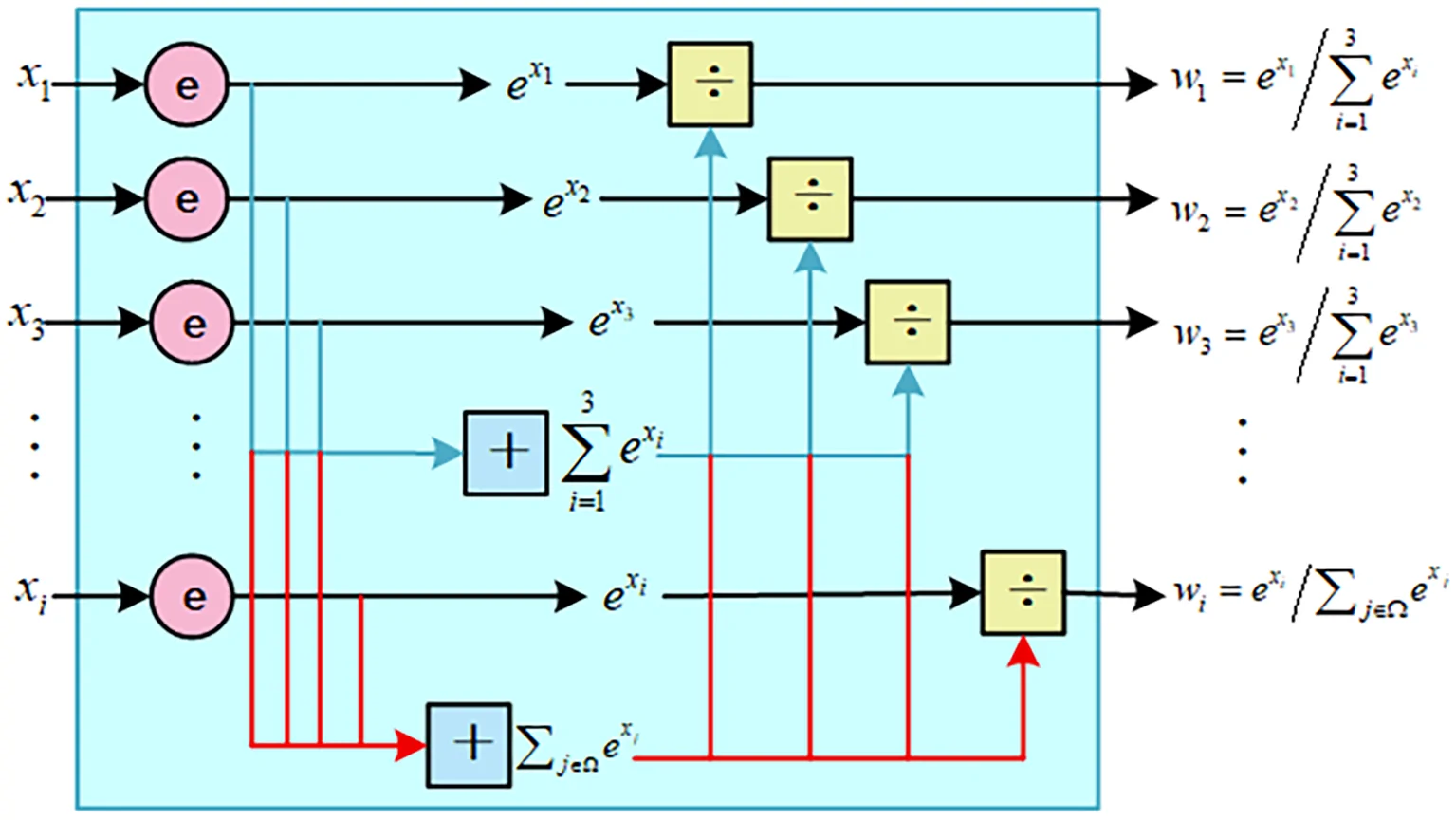
Architecture of the softmax module.

Using these weights, the output of soft pooling, denoted *y_soft_*, is defined as the weighted sum of local features within the receptive field as defined in Equation 2-8:

(2-8) $$y_{soft}=\sum \left(w_{i}\cdot x_{i}\right),x_{i}\in \Omega$$

While the dual branches extract complementary local features, different channels in deep networks encode diverse semantic information and thus exhibit varying requirements for sharp details and smooth contextual information. To achieve channel-wise adaptive adjustment, GHP incorporates a lightweight gating sub-branch. First, the input feature map X is compressed into a channel descriptor via Global Average Pooling (GAP). A 1×1 convolutional layer followed by a Sigmoid activation function σ, then generates the channel-wise gating vector as expressed in Equation 2-9 $g\in {\text{R}}^{C\times 1\times 1}$:

(2-9) g=σ(Conv(GAP(X)))

Where the weights and biases of the 1×1 convolution serve as trainable parameters of the network. Each element g∈(0,1) in the gating vector g reflects the preference degree of the c-th channel for MaxPool features, whereas its complementary term (1-g) indicates the corresponding preference for SoftPool features. The two branches are fused adaptively, as expressed in Equation 2-10

(2-10) $$GHP\left(X\right)=g\cdot y_{\max}+\left(1-g\right)\cdot y_{soft}$$

Since g+(1−g)=1 holds for each channel, this fusion scheme forms a mathematically dynamic convex combination, thereby ensuring numerical stability of the output magnitude. When g → 1, the corresponding channel reduces to standard MaxPool; when g → 0, it equivalently reduces to SoftPool.

To address the pronounced scale variations of rice diseases, this study proposes the GHP-SPPF module to replace the original SPPF module in YOLOv8. Structurally, the module replaces the original 5×5 max pooling kernel with the proposed GHP module while maintaining the same receptive field of k=5, ensuring that the difference between the two branches arises solely from the pooling mechanism. Mechanistically, GHP runs parallel MaxPool and SoftPool branches: the MaxPool branch captures high-frequency responses to small lesions, while the SoftPool branch preserves edge gradient information of large lesions. Building on this design, GHP-SPPF incorporates a channel-wise learnable gating mechanism, replacing the conventional fixed hard selection with adaptive fusion. GHP-SPPF introduces additional computation due to the parallel SoftPool branch and channel-wise gating operation. Therefore, its computational cost is quantitatively evaluated in terms of parameters, FLOPs and peak GPU memory in the experimental section.

### 2.4 Channel-adaptive feature fusion module

To improve cross-scale feature fusion, BiFPN (Tan et al., 2020) assigns learnable scalar weights to each input branch, while Gold-YOLO (Wang et al., 2023) enables global information aggregation across pyramid levels. Subsequent studies such as CCFM (Chen et al., 2026) and MSAFF (Tian et al., 2026) introduce channel compression and attention recalibration after concatenation; however, these methods either weight features only at branch granularity or refine them after equal-weighted fusion, leaving channel-adaptive pre-concatenation weighting unexplored.

In the PAN-FPN (Liu et al., 2018) neck of YOLOv8, cross-scale features are directly concatenated along the channel axis via Concat and fed into the C2f module. However, this static concatenation assumes equal channel contributions and neglects cross-level scale discrepancies, leading to gradient imbalance and impaired convergence. To address these limitations, this study proposes the Channel-Adaptive Feature Fusion (CAFF) module, which integrates learnable channel-wise weighting, distribution alignment, and residual refinement to achieve precise cross-level fusion for multi-scale targets.

As illustrated in **Figure 6**, given two input feature maps $X_{1}\in {\text{R}}^{C_{1}\times H\times W}$ and $X_{2}\in {\text{R}}^{C_{2}\times H\times W}$, the module introduces trainable weight tensors $w_{1}\in {\text{R}}^{C_{1}}$ and $w_{2}\in {\text{R}}^{C_{2}}$ respectively. The weights are normalized via the Sigmoid function and then applied to perform channel-wise weighting on the input features, as formulated in Equation 2-11:

**Figure 6:**
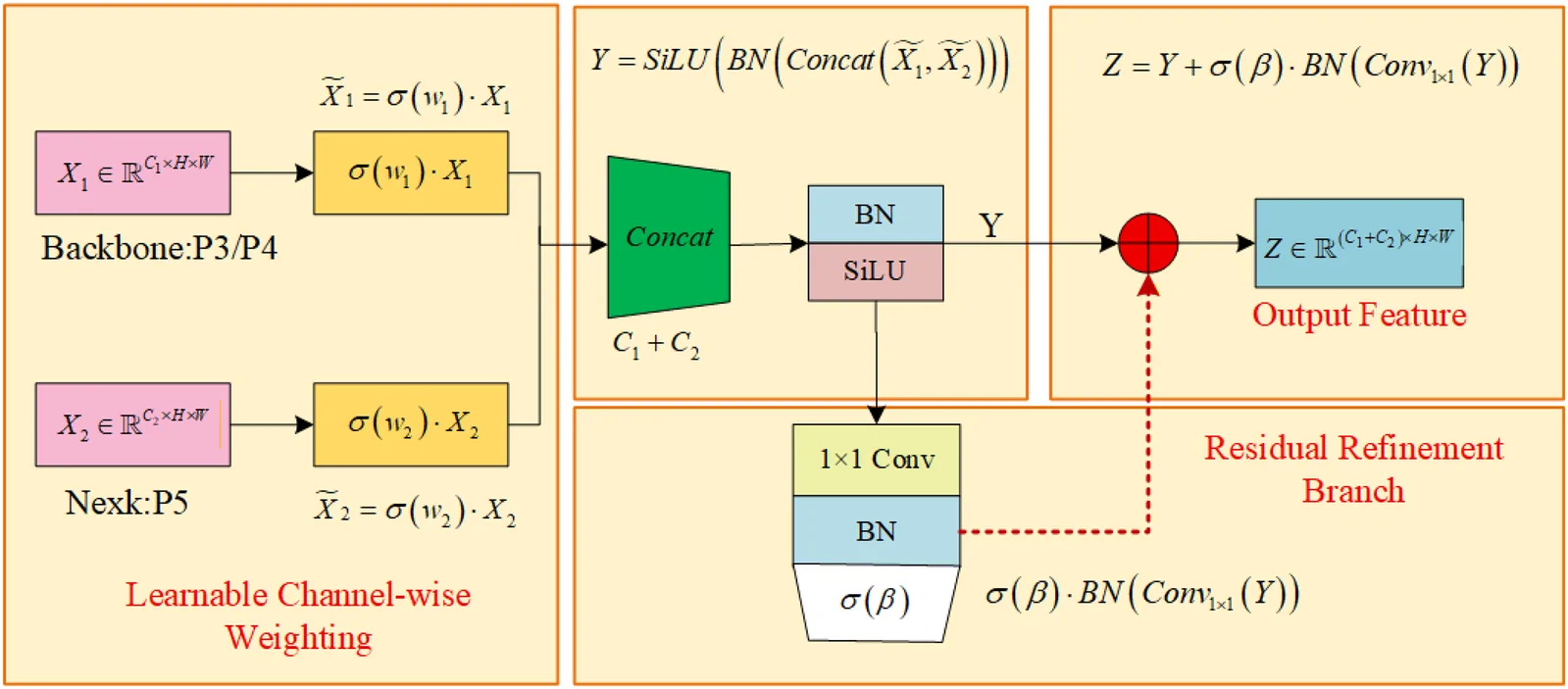
Architecture of the CAFF module.

(2-11) $$\{\begin{array}{c} X_{1}=\sigma \left(w_{1}\right)\cdot X_{1}\\ X_{2}=\sigma \left(w_{2}\right)\cdot X_{2} \end{array}$$

Following concatenation of the weighted features ${\tilde{X}}_{1}$ and ${\tilde{X}}_{2}$ along the channel axis, a batch normalization layer is employed to align their feature distributions, thereby eliminating discrepancies in feature response scale distributions as shown in Equation 2-12 between shallow and deep features:

(2-12) $$Y=BN\left[Concat\left(X_{1},X_{2}\right)\right]$$

The concatenated features are then passed through the SiLU activation function to enhance representational capacity. With both smooth and non-monotonic properties, SiLU enables flexible modeling of complex relationships among features as expressed in Equation 2-13:

(2-13) $$Y_{act}=SiLU\left(Y\right)$$

Furthermore, to further improve fusion quality, CAFF incorporates a gated residual refinement sub-branch. This branch extracts residual features from the fused output via a 1×1 convolution and BN layer, which are then modulated by a trainable gating parameter *β* and as formulated in Equation 2-14:

(2-14) $$Z=Y_{act}+\sigma \left(\beta \right)\cdot BN\left(Conv_{1\times 1}\left(Y_{act}\right)\right)$$

Specifically, the trainable residual-gating parameter *β* is initialized to -3.0. After the Sigmoid transformation, the initial gate value is σ(−3)≈0.047, the residual refinement branch contributes only a small correction at the beginning of training. As training proceeds, *β* jointly optimized with the network parameters to adaptively adjust the contribution of residual refinement.

This study uses CAFF to replace all Concat operations in the PAN-FPN neck of YOLOv8s. For early-stage small lesions such as rice blast, the module automatically enhances edge and texture channels in shallow high-resolution features, preventing subtle lesion representations from being overshadowed by deep semantic information. By contrast, for large lesions such as bacterial leaf blight, the model assigns higher weights to deep global context channels. At each fusion stage, shallow edge details and deep semantic information are independently recalibrated channel-wise before fusion. This design effectively addresses the inherent limitation of conventional static Concat, which fails to differentiate the contributions of individual channels. The proposed approach provides a more flexible and robust feature fusion framework for multi-scale crop disease detection, leading to notable improvements in detection accuracy and generalization.

## 3 Results and discussion

### 3.1 Experimental environment and setup

All experiments were performed on an Ubuntu server equipped with an NVIDIA GeForce RTX 3090 GPU (24 GB). The software environment included Python 3.10, PyTorch 2.5.1, and CUDA 12.1, and the detection framework was built on Ultralytics YOLO 8.4.82. The model was trained using the stochastic gradient descent (SGD) optimizer with a momentum of 0.937, an initial learning rate of 0.01, and a weight decay of 5×10⁻^4^. The batch size was set to 32, all input images were resized to 640×640 pixels, and the model was trained for 150 epochs.

### 3.2 Experimental dataset

In this study, the rice disease detection dataset is sourced from the Roboflow Universe open-source platform (Rice Disease Dataset, Roboflow Universe). Designed for rice leaf disease detection under authentic field conditions, this dataset encompasses three prevalent rice diseases, namely Rice Bacterial Blight, Rice Blast, and Rice Brownspot. The dataset is divided into three subsets, containing 934, 267, and 134 images for training, validation, and testing, respectively. All images are captured from natural field environments and involve complex field interference factors, including illumination variations, leaf occlusion, weeds, and soil backgrounds, thereby rendering the dataset consistent with practical application scenarios. Representative disease samples are illustrated in **Figure 7**.

**Figure 7:**
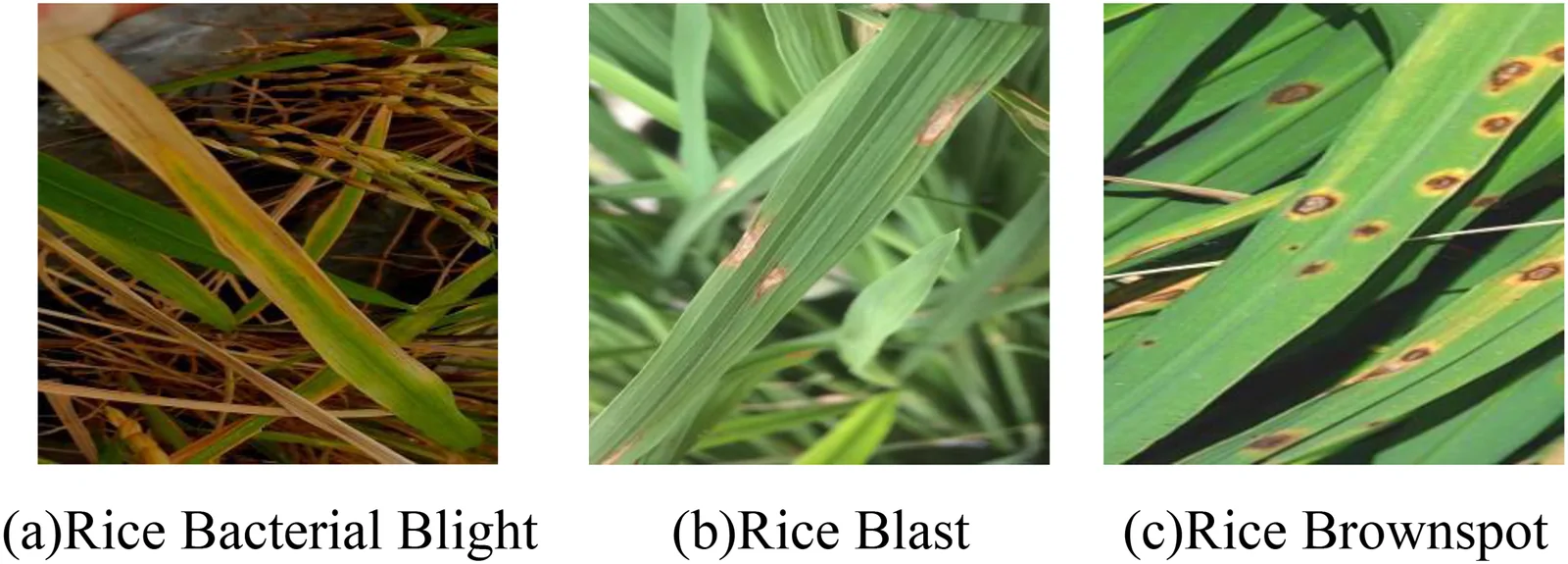
Typical samples of the rice disease dataset: **(a)** Rice Bacterial Blight, **(b)** Rice Blast, and **(c)** Rice Brownspot.

As depicted in **Figure 8**, the logarithmic distribution of bounding box count and average box area per image in the training set is presented using a scatter bubble chart, with the three disease categories distinguished by color and shape. The three diseases form clearly separated clusters, revealing notable differences in object density and scale: rice bacterial blight (blue) clusters on the left, exhibiting a “sparse large object” pattern in which most images contain only one lesion with large box areas; rice brown spot (red) concentrates in the lower-right region, exhibiting a “dense small object” pattern with a high average number of bounding boxes per image and small box sizes; and rice blast (green) falls in between, with moderate box count and area. Furthermore, only a limited number of images contain annotations of two or more disease categories. This scarcity of cross-class co-occurrence hinders the model from learning the mixed distribution of multiple diseases, rendering it prone to relying on global context while neglecting fine-grained lesion discrimination. Such dual imbalance in both sample size and spatial distribution poses significant challenges for detection models, necessitating targeted data augmentation strategies.

**Figure 8:**
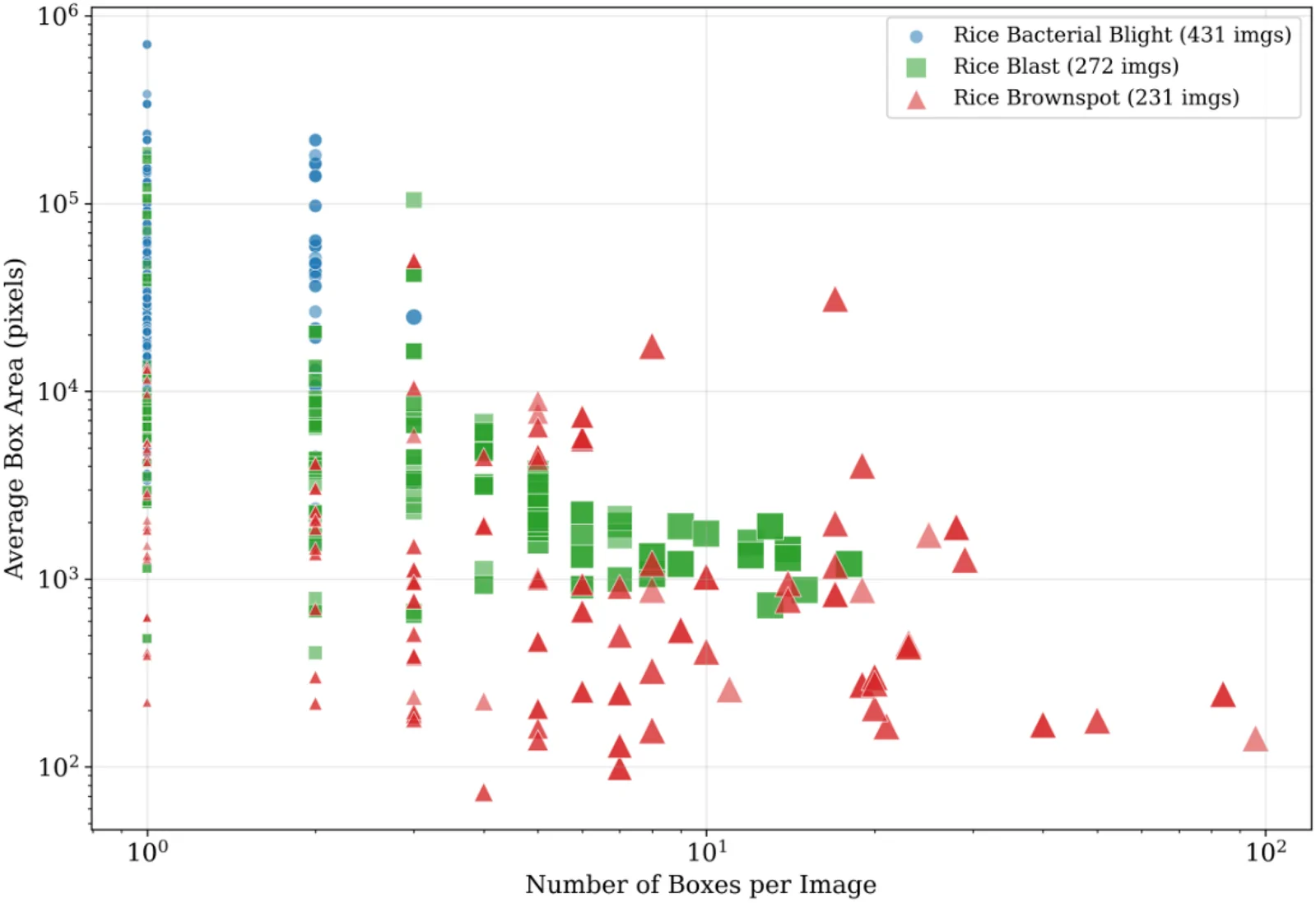
Statistical distribution of per-image annotations in the training set.

The primary dataset is relatively limited in size, particularly the test subset containing 134 images. Therefore, the results on this dataset should not be interpreted as definitive evidence of universal superiority. To reduce the influence of stochastic training variation, repeated experiments with multiple random seeds are additionally reported, and two independent public datasets are used to examine cross-dataset behavior.

### 3.3 Evaluation metrics

To quantitatively evaluate the performance of the improved network, the following metrics are employed as the final evaluation benchmarks: Precision (P) (Equation 3-1, Recall (R) (Equation 3-2), F1-score (F1) (Equation 3-3), average precision (AP) (Equation 3-4), mAP@0.5 (Equation 3-5), and mAP@0.5:0.95 (Equation 3-6).

(3-1) $$P=\frac{TP}{TP+FP}$$

(3-2) $$R=\frac{TP}{TP+FN}$$

(3-3) $$F_{1}=\frac{2\times P\times R}{P+R}$$

(3-4) $$AP=\int_{0}^{1}P\left(R\right)dR$$

(3-5) $$mAP50=\frac{\sum_{j=1}^{k}AP_{i}}{k\left(classes\right)}\left(IOU=0.5\right)$$

(3-6) $$mAP95=\frac{\sum_{j=1}^{k}\sum_{t=0}^{9}AP_{i}}{10k\left(classes\right)}\left(IOU=0.5+0.05t\right)$$

### 3.4 Experimental results

To validate the effectiveness of the proposed AGC-YOLO method, comparative experiments are performed against the baseline YOLOv8s on the rice leaf disease dataset with identical experimental settings. Experimental results demonstrate that AGC-YOLO achieves a mean Average Precision (mAP) of 93.5 ± 0.6%, representing a 3.0 percentage-point improvement over the baseline model. Detailed training results are illustrated in **Figure 9**.

**Figure 9:**
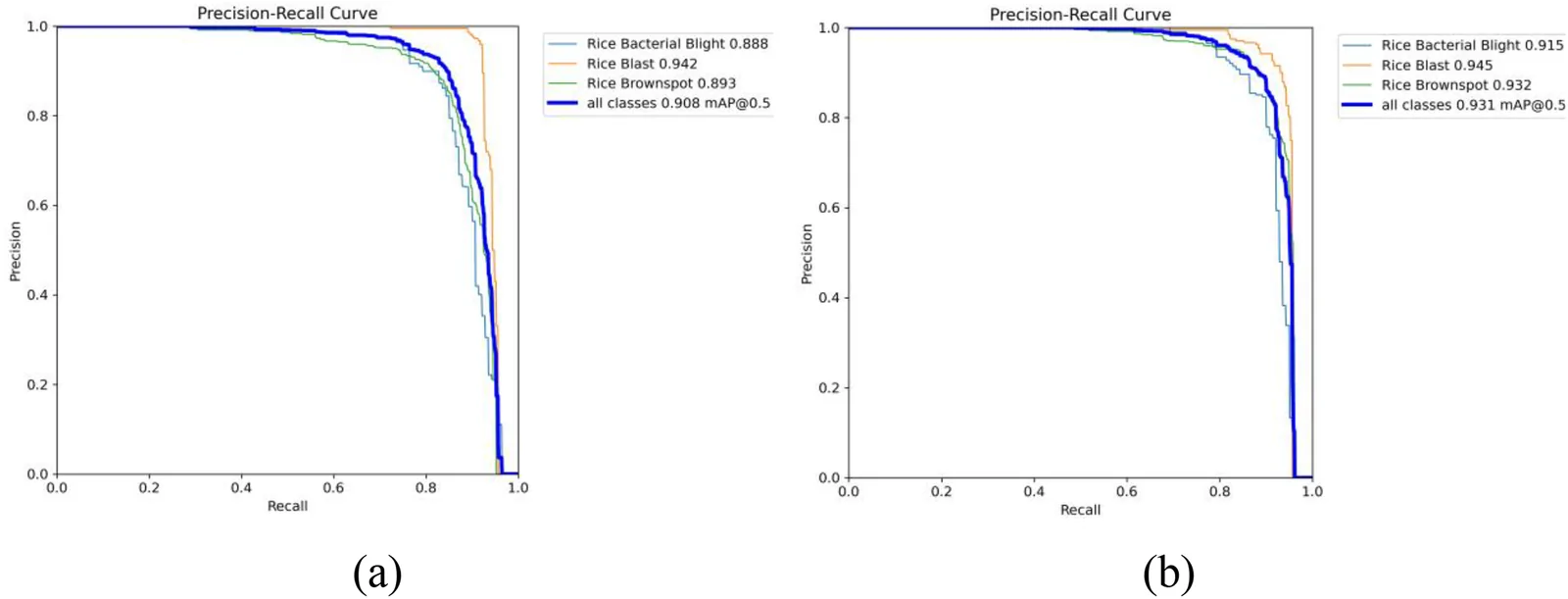
Precision–Recall curves on the rice validation set for a representative run (random seed 0): **(a)** YOLOv8s baseline; **(b)** the proposed AGC-YOLO.

### 3.5 Ablation experiment

To evaluate the contribution and robustness of the proposed modules, ablation experiments were conducted on the rice disease dataset. YOLOv8s trained on the original dataset was used as the baseline, while SAICP Net, GHP-SPPF, and CAFF were progressively introduced. All experiments were performed under the same training configuration, and each configuration was independently repeated using five random seeds (0–4). The results are reported as mean ± standard deviation in **Table 2**.

**Table 2:** Results of ablation experiments.

Model				P (%)	R (%)	mAP_50_ (%)	mAP_50-95_ (%)	Params/M	GFLOPS
Baseline	SAICP net	GHP_SPPF	CAFF						
✓				91.3±1.4	84.9±1.1	90.5±0.9	62.1±1.3	11.14	28.4
✓	✓			92.6±1.3	87.4±1.0	92.0±0.8	66.7±0.4	11.14	28.4
✓	✓	✓		93.6±1.0	87.3±0.9	92.4±0.4	68.5±0.9	11.20	28.6
✓	✓	✓	✓	93.2±0.8	87.8±0.4	93.5±0.6	69.1±0.8	12.68	33.4

Compared with the baseline YOLOv8s, introducing SAICP Net increased mAP@0.5 from 0.905 ± 0.009 to 0.920 ± 0.008, corresponding to an improvement of 1.5 percentage points. These results indicate that the semantic-aware augmentation strategy provides a consistent improvement without increasing the model parameters or computational complexity. On the basis of SAICP Net, the introduction of GHP-SPPF further increased mAP@0.5 to 0.924 ± 0.004 and mAP@0.5:0.95 to 0.685 ± 0.009. The parameter count increased only from 11.14 M to 11.20 M, while the computational complexity cost increased slightly from 28.4 to 28.6 GFLOPs. This result indicates that GHP-SPPF provides an additional improvement in multi-scale feature representation with relatively limited computational overhead. After integrating all three modules, AGC-YOLO attained an mAP@0.5 of 93.5 ± 0.6%, representing a 3.0 percentage-point improvement over the baseline without performance degradation, thereby validating the functional complementarity and synergistic enhancement among the three components.

### 3.6 Performance comparison of different detection algorithms

In order to verify the detection performance of AGC-YOLO, this study contrasted it with the two-stage detector Faster R-CNN (Ren et al., 2016) and a range of one-stage algorithms, including RT-DETR (Zhao et al., 2024), YOLOv5s, YOLOv8s, YOLOv9s (Wang et al., 2024), YOLOv10s (Wang et al., 2024), YOLOv11s, and YOLOv26s, along with two recently proposed counterpart models, YOLO-DP and MGA-YOLO. All models were trained and evaluated under the same data split and training budget, with SAICP Net augmentation applied uniformly and the random seed fixed at 0 for all comparison experiments. No extensive model-specific hyperparameter search was performed; the comparison was conducted under a common data split and training budget to provide a controlled within-study comparison. The results are reported in **Table 3**.

**Table 3:** Comparative experimental results with other models.

Model	P (%)	R (%)	mAP_50_ (%)	mAP_50-95_ (%)	F1-score
YOLOv5s	92.1	84.5	91.5	64.7	0.88
YOLOv8s	93.2	86.6	91.5	68.5	0.88
YOLOv9s	87.0	81.1	86.6	56.5	0.84
YOLOv10s	86.3	82.8	88.4	59.0	0.84
YOLOv11s	91.3	86.2	90.4	63.0	0.89
YOLOv26s	83.6	79.4	85.9	51.9	0.81
RT-DETR	81.0	58.1	68.4	44.9	0.67
Faster-RCNN	32.1	77.0	77.8	41.4	0.45
YOLO-DP	91.9	86.2	90.6	63.6	0.89
MGA-YOLO	91.2	81.9	89.3	60.3	0.86
AGC-;YOLO	91.6	87.2	93.1	69.1	0.89

The results are reported in **Table 3**, which shows that AGC-YOLO achieves state-of-the-art performance, with mAP@0.5 and mAP@0.5:0.95 reaching 93.1% and 69.1%, respectively, which are 1.6% and 0.6 percentage points higher than those of the baseline YOLOv8s. In comparison with the aforementioned models, the mAP@0.5 of AGC-YOLO is respectively 1.6%, 1.6%, 6.5%, 4.7%, 2.7%, 7.2%, 24.7%, 15.3%, 2.5%, and 3.8% higher than those of YOLOv5s, YOLOv8s, YOLOv9s, YOLOv10s, YOLOv11s, YOLOv26s, RT-DETR, Faster R-CNN, YOLO-DP, and MGA-YOLO. These results demonstrate that SAICP Net provides semantically compliant and diverse training samples, GHP-SPPF enhances the Backbone’s feature retention capability for multi-scale lesions, and CAFF improves cross-scale feature fusion in the Neck, whose synergistic effect enables AGC-YOLO to outperform counterpart methods in both accuracy and robustness. Hence, AGC-YOLO can be regarded as an effective approach for rice disease detection.

### 3.7 Cross-dataset generalization experiments

To comprehensively validate the cross-domain generalization capability of AGC-YOLO, two publicly available agricultural datasets with notable visual challenges were incorporated as supplementary test sets. The objective is to evaluate the model’s generalization to fine-grained lesion recognition under high inter-class morphological similarity.

1. Tomato Leaf Diseases Dataset: This public benchmark for tomato leaf disease detection is published on the Roboflow platform (Tomato Leaf Diseases Detection Dataset, Roboflow Universe). It encompasses seven categories, namely Bacterial Spot, Early Blight, Late Blight, Leaf Mold, Target Spot, Black Spot, and Healthy. All images are uniformly resized to 640×640 pixels with annotations in YOLO format. This dataset is employed to validate the cross-crop generalization robustness of AGC-YOLO under diverse lesion morphologies and complex leaf backgrounds.
2. Rice Leaf Dataset: This public dataset for rice leaf disease detection is published by UtopiaLab on the Roboflow platform (Rice Leaf Dataset, Roboflow Universe). It comprises 5,188 images across 11 categories, including Brown Spot, Leaf Blight, Leaf Scald, Leaf Blast, Leaf Smut, Narrow Brown Spot, Rice Bacterial Blight, Rice Blast, Rice Brown Spot, Bacterial Leaf Blight, and Healthy. The dataset contains multiple fine-grained disease pairs with high visual similarity, such as Brown Spot and Narrow Brown Spot, which poses considerable challenges to the inter-class discriminative capability of detection models. This dataset is adopted to validate the robustness of AGC-YOLO in multi-category fine-grained disease detection scenarios.

First, comparative experiments across multiple mainstream detection models were performed on the Tomato Leaf Diseases dataset, with results summarized in **Table 4**. AGC-YOLO attains an mAP@0.5 of 76.3%, representing a 3.1 percentage-point improvement over the baseline YOLOv8s, and achieves the highest precision of 82.9% among all comparative models, thereby verifying that the improved Neck feature fusion and pooling strategies effectively mitigate the interference induced by complex tomato leaf textures. By contrast, Faster R-CNN yields an mAP@0.5 of only 41.7%, reflecting the pronounced limitations of two-stage detectors on small-scale agricultural datasets. Notably, YOLO-DP also attains an mAP@0.5 of 76.3%, yet its precision (77.7%) lags behind that of AGC-YOLO (82.9%), suggesting that the channel-adaptive weighting mechanism of CAFF delivers tangible gains in reducing background false positives under cross-crop scenarios. Collectively, these results demonstrate that the architectural improvements of AGC-YOLO exhibit no dependency on specific crop types, thereby endowing the model with favorable cross-domain transfer capability.

**Table 4:** Comparative experimental results on the tomato leaf diseases dataset.

Model	P (%)	R (%)	mAP_50_ (%)	mAP_50-95_ (%)	F1-score
YOLOv5s	74.1	70.0	73.3	47.2	0.70
YOLOv8s	74.9	73.1	73.2	48.9	0.73
YOLOv9s	81.1	68.6	74.5	48.5	0.73
YOLOv10s	66.3	65.6	65.7	44.7	0.64
YOLOv11s	77.1	68.4	71.7	47.0	0.71
YOLOv26s	60.5	68.2	66.1	43.8	0.63
Faster-RCNN	21.2	44.6	41.7	25.6	0.29
YOLO-DP	77.7	77.1	76.3	48.6	0.76
MGA-YOLO	78.4	72.2	75.6	47.7	0.74
AGC-;YOLO	82.9	74.4	76.3	48.8	0.77

To further verify the robustness of AGC-YOLO under multi-class fine-grained disease scenarios, comparative experiments were performed on the Rice Leaf dataset covering 11 rice leaf disease categories, with results detailed in **Table 5**. This dataset contains approximately four times the number of categories compared with the original rice disease dataset and features multiple pairs of visually similar fine-grained diseases, such as Brown Spot versus Narrow Brown Spot and Rice Blast versus Leaf Blast, thereby imposing higher demands on the inter-class discriminative capability of detection models. AGC-YOLO attains the highest mAP@0.5 of 57.0% and recall of 59.9%, representing improvements of 1.3% and 0.9%, respectively, over the baseline YOLOv8s. Although the absolute improvement margin is diminished relative to that on the three-class rice disease dataset, which is primarily attributed to the scarce annotations of certain categories and the minute inter-class discriminative features, AGC-YOLO achieves the highest mAP@0.5, mAP@0.5:0.95, recall, and F1-score among all comparative models, thereby verifying the effectiveness and stability of the three-module synergistic design in multi-class fine-grained detection scenarios.

**Table 5:** Comparative experimental results on the rice leaf dataset.

Model	P (%)	R (%)	mAP_50_ (%)	mAP_50-95_ (%)	F1-score
YOLOv5s	55.5	56.9	55.5	34.2	0.55
YOLOv8s	55.8	59.0	55.7	35.4	0.56
YOLOv9s	58.8	57.1	56.2	35.5	0.57
YOLOv10s	54.9	56.7	54.6	34.9	0.54
YOLOv11s	54.4	58.6	56.9	36.0	0.56
YOLOv26s	54.4	53.5	54.0	32.3	0.53
Faster-RCNN	15.7	58.1	43.4	21.6	0.25
YOLO-DP	56.7	58.0	56.5	35.7	0.56
MGA-YOLO	53.3	57.4	55.6	34.3	0.55
AGC-;YOLO	58.5	59.9	57.0	36.7	0.58

### 3.8 Baseline model selection

In order to further evaluate the applicability of YOLOv8s as the baseline model, this study trained four YOLOv8 variants, namely YOLOv8n, YOLOv8s, YOLOv8m, and YOLOv8l, on the same rice disease dataset. The training outcomes for each model are presented in **Table 6**.

**Table 6:** Comparative experimental results of different YOLOv8 versions.

Model	P (%)	R (%)	mAP_50_ (%)	mAP_50-95_ (%)	Params/M
YOLOv8n	84.2	79.7	86.1	50.3	3.00
YOLOv8s	92.5	87.4	90.8	63.0	11.14
YOLOv8m	91.8	86.7	92.2	67.8	25.84
YOLOv8l	87.7	85.5	88.8	63.0	43.61

The experimental findings show that the network’s width and depth are positively correlated with overall detection accuracy, and the parameter count grows proportionally with the number of layers. However, YOLOv8l, with 43.61 million parameters, exhibits a reduced mAP@0.5 of 88.8%, indicating that on agricultural datasets with limited samples, may indicate that additional capacity does not translate into improved performance on this dataset and thereby constrains generalization capability. YOLOv8n has the fewest parameters, but its mAP, precision, and recall are all lower than those of the other variants, falling short of the accuracy requirements for disease detection in complex paddy field environments. In contrast, YOLOv8m achieves a 1.4% higher mAP@0.5 than YOLOv8s but incurs a 132% increase in parameters, representing a performance gain far outweighed by the parameter overhead. By comparison, YOLOv8s achieves a practical trade-off between detection accuracy and computational efficiency with only 11.14 million parameters. Therefore, after a thorough evaluation of these factors, this study selected YOLOv8s as the baseline model.

### 3.9 Visualization result analysis

In order to visually interpret the attention distributions of different models, this study employed ScoreCAM (Wang et al., 2020a) to conduct a comparative visualization between the baseline YOLOv8s and AGC-YOLO across the three datasets. ScoreCAM quantifies the contribution of each feature channel to the target category via channel-wise forward perturbation; as a gradient-free method, it mitigates the gradient dilution and peak diffusion issues inherent in Grad-CAM (Selvaraju et al., 2017) and Grad-CAM++ (Chattopadhay et al., 2018) under dense small-target scenarios. In the heatmaps, attention intensity is represented by a color gradient from red (high) to blue (low). As illustrated in **Figures 10**–**12**, AGC-YOLO generally exhibits more concentrated responses around lesion-related regions in the selected examples, with reduced activation in some background regions. On the primary rice disease dataset, the AP@0.5 values of bacterial leaf blight, rice blast, and rice brown spot increase from 0.888, 0.942, and 0.893 to 0.915, 0.945, and 0.932, respectively, while the overall mAP@0.5 increases from 0.908 to 0.931.

**Figure 10:**
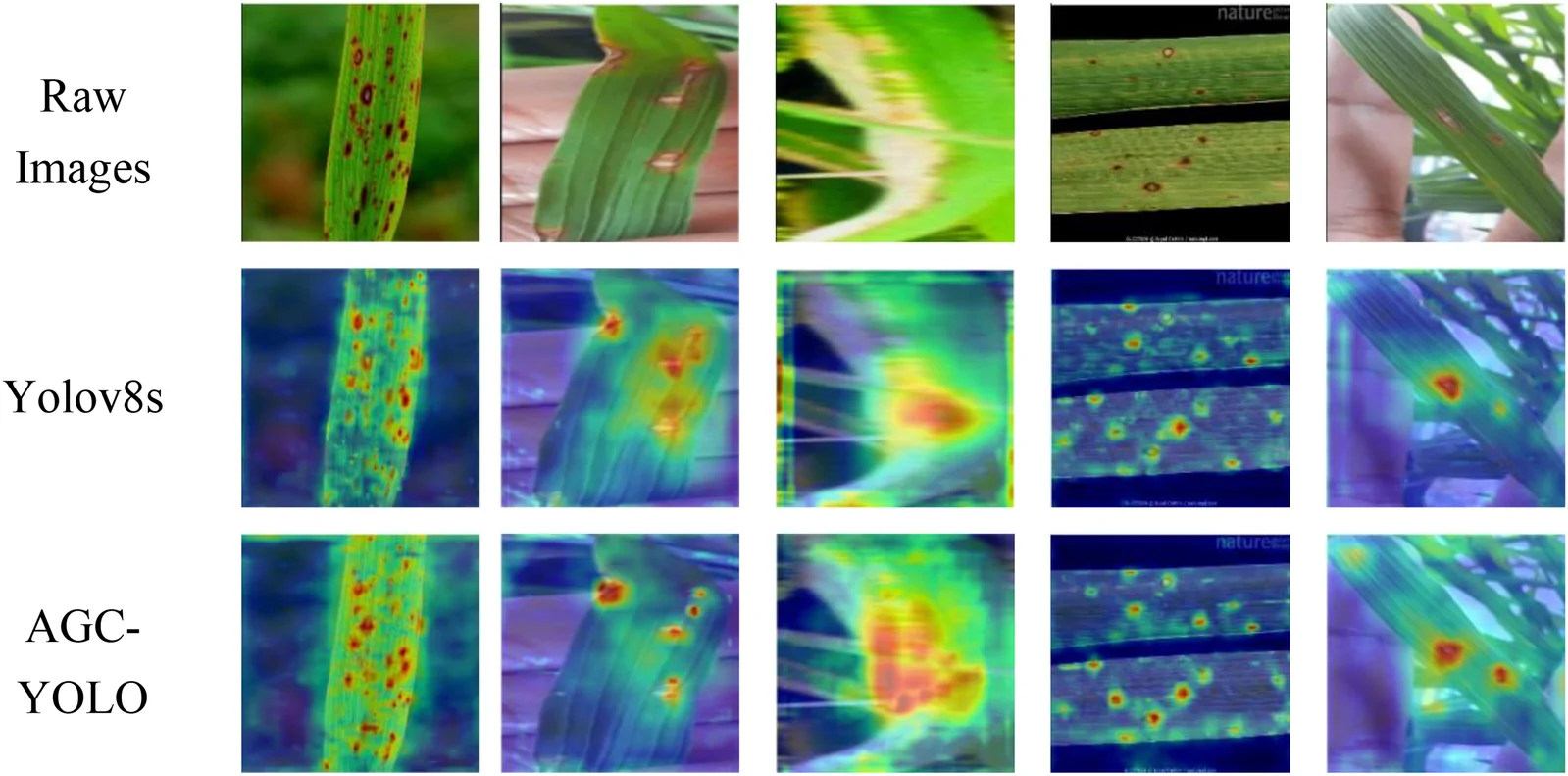
Heatmap of rice disease dataset.

**Figure 11:**
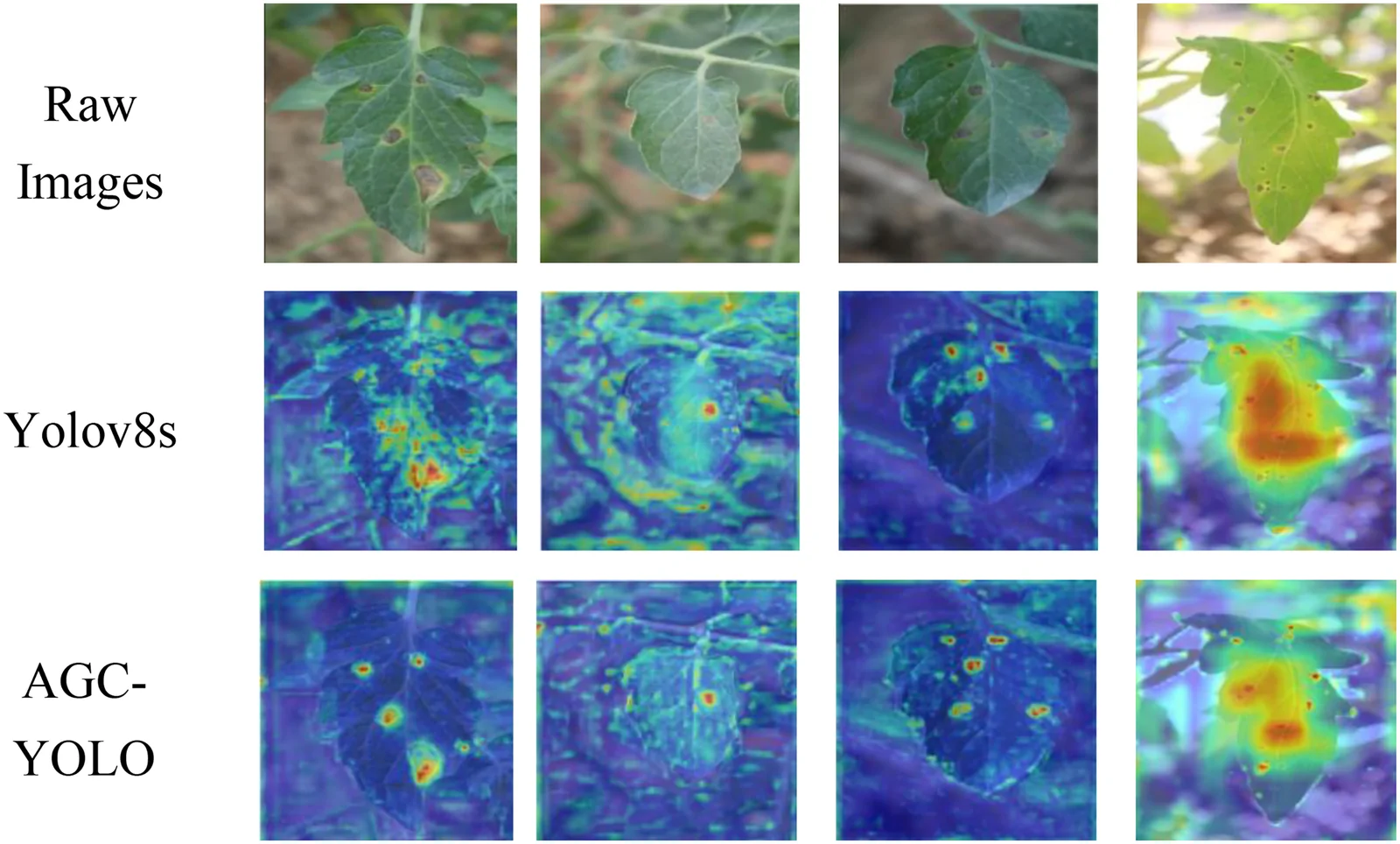
Heatmap of tomato leaf disease dataset.

**Figure 12:**
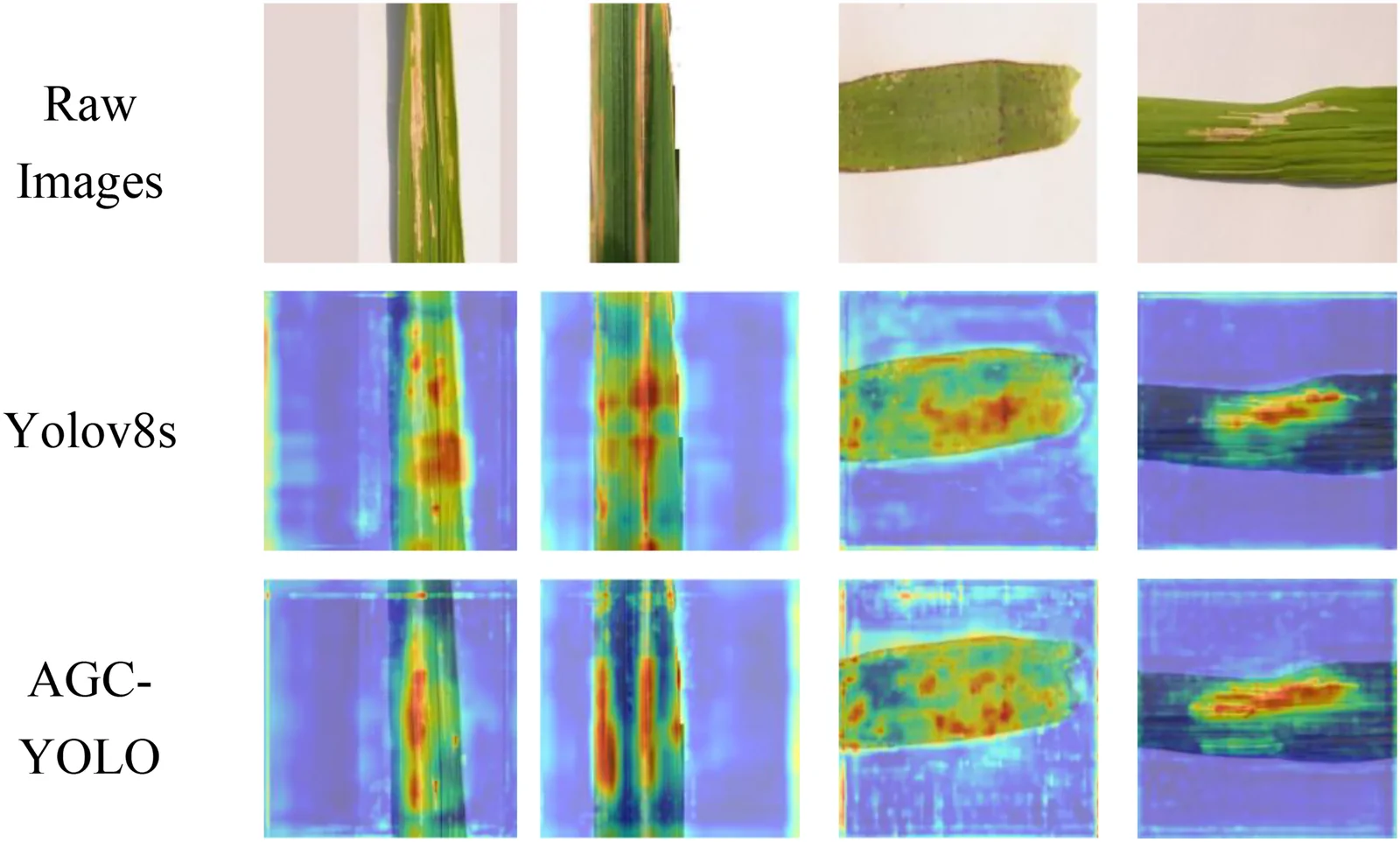
Heatmap of rice leaf disease dataset.

The normalized confusion matrices in **Figures 13**–**15** further reveal the remaining detection errors. On the primary rice disease dataset, the proportion of rice brown spot samples assigned to background decreases from 0.11 to 0.06, while its correct classification rate increases from 0.89 to 0.94. Similar reductions in background-related missed detections are observed for bacterial leaf blight (0.11 to 0.10) and rice blast (0.07 to 0.06).

**Figure 13:**
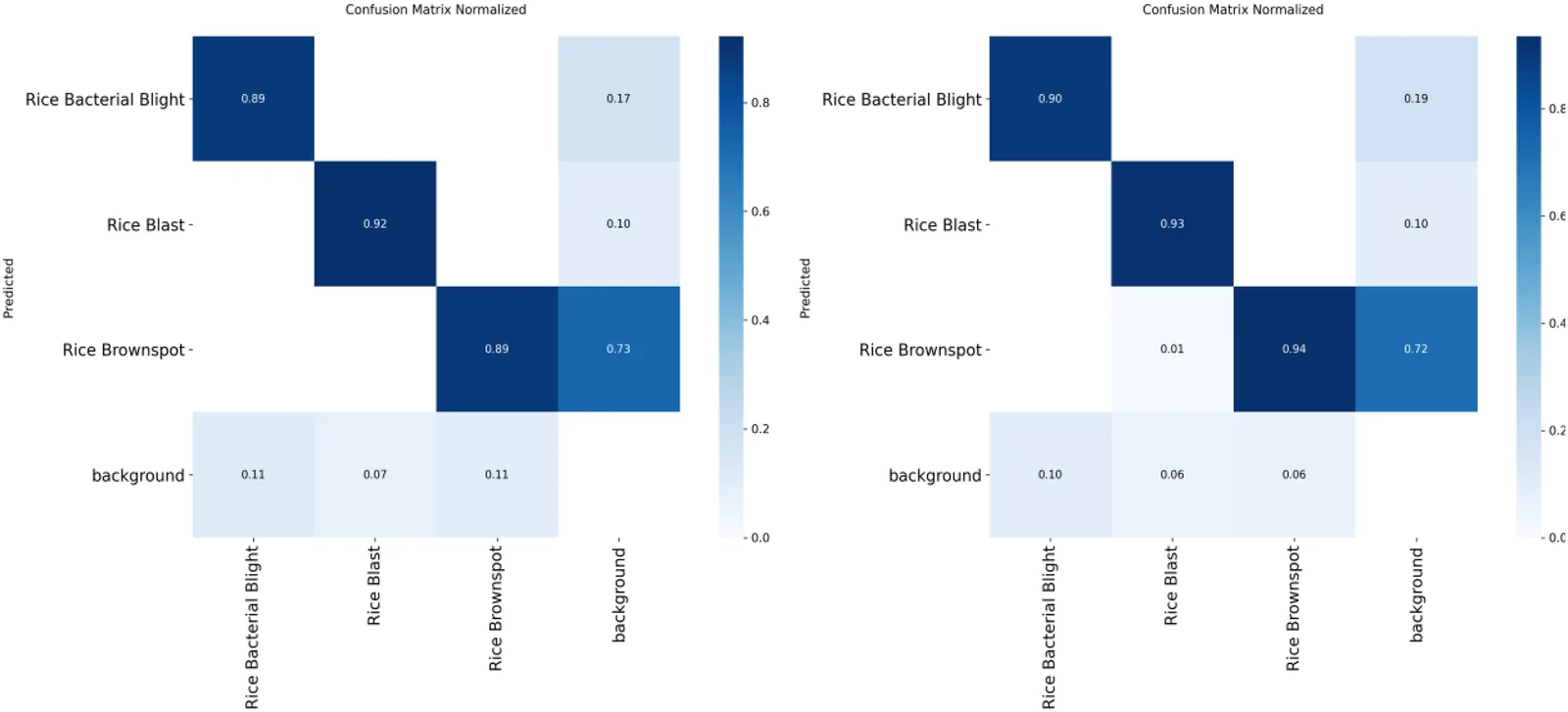
Confusion matrix comparison on the rice disease dataset: YOLOv8s vs. AGC-YOLO.

**Figure 14:**
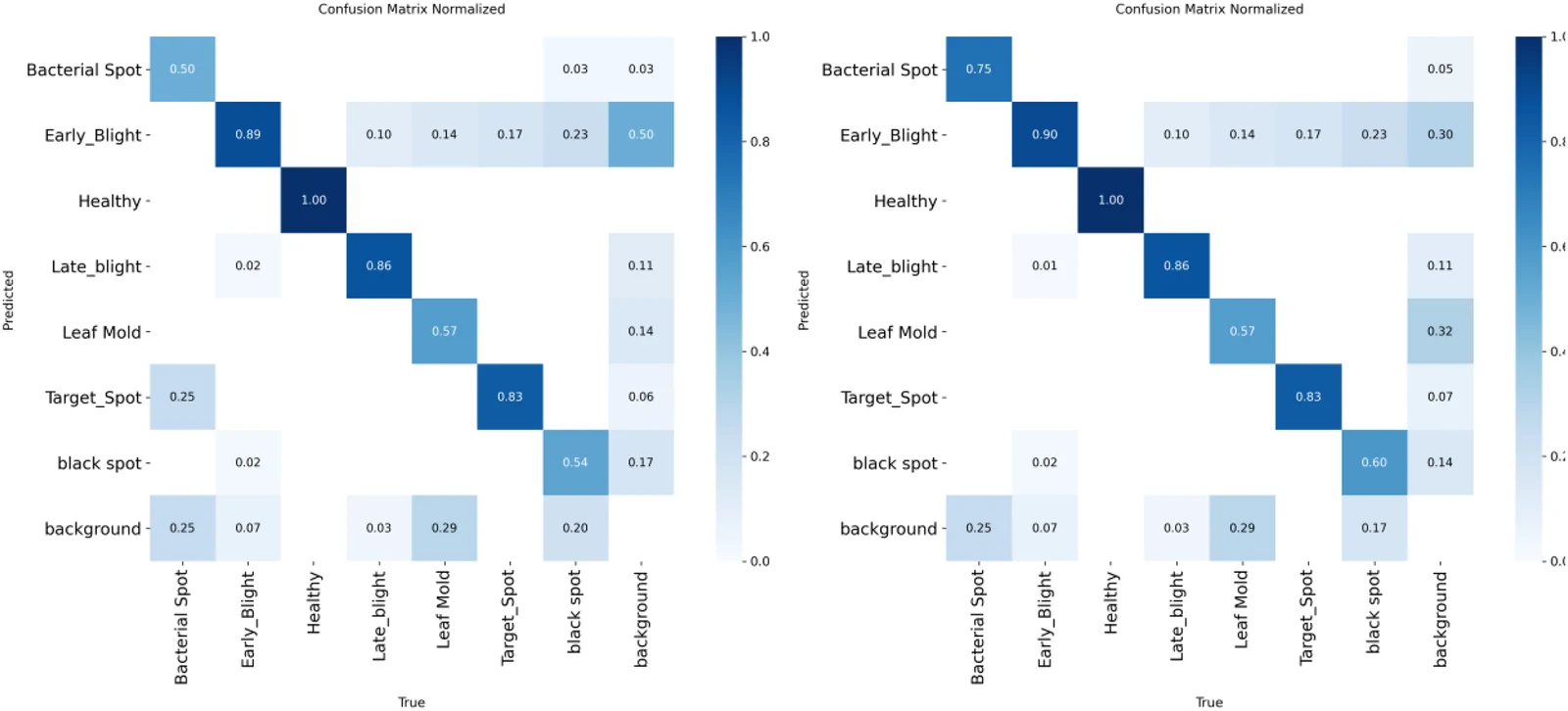
Confusion matrix comparison on the tomato leaf diseases dataset: YOLOv8s vs. AGC-YOLO.

**Figure 15:**
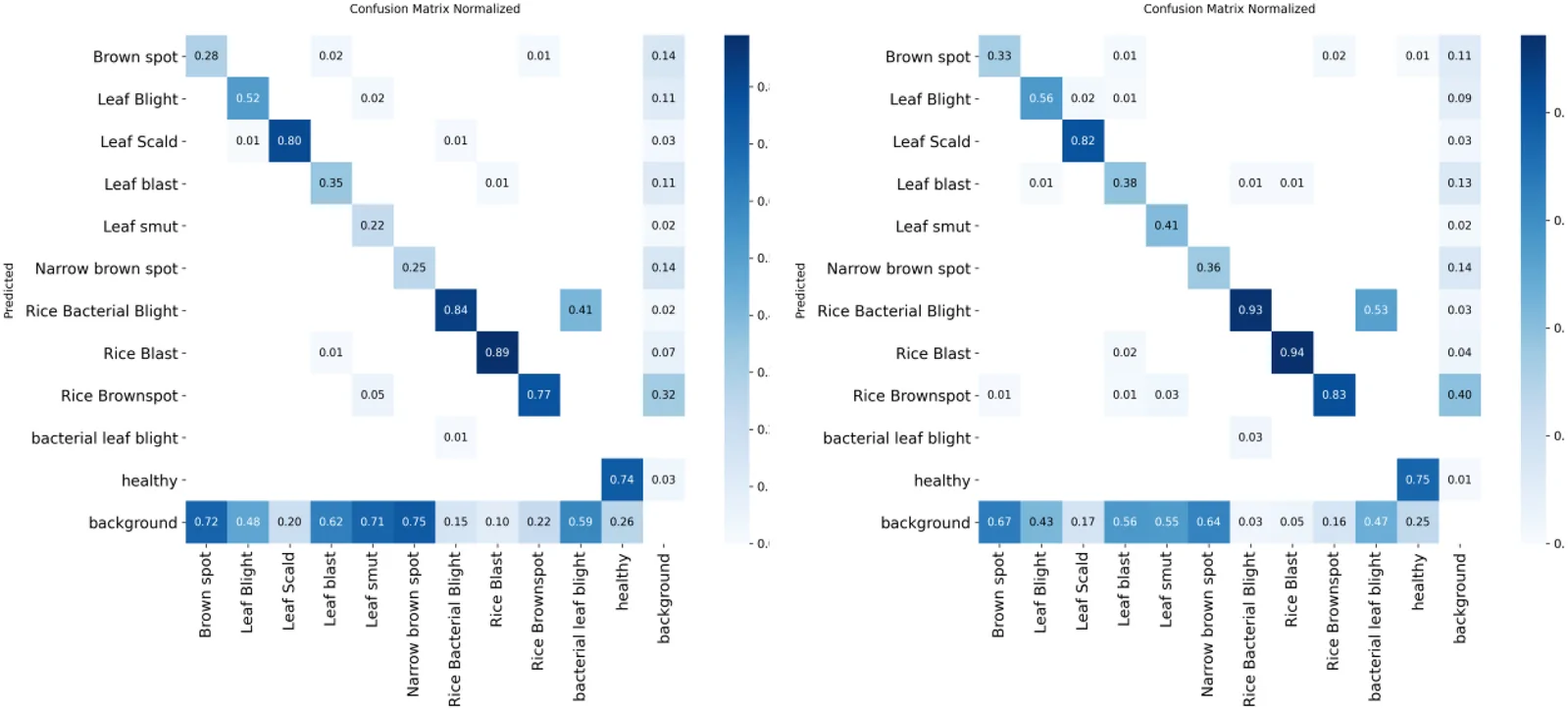
Confusion matrix comparison on the rice leaf dataset: YOLOv8s vs. AGC-YOLO.

For the Tomato Leaf Diseases dataset, the most evident improvement occurs for Bacterial Spot, whose diagonal value increases from 0.50 to 0.75, while its confusion with Target Spot is reduced. Black spot also increases from 0.54 to 0.60. Nevertheless, background-related false detections remain for several categories, particularly Leaf Mold, indicating that complex background patterns can still activate disease-related responses. On the 11-class Rice Leaf dataset, missed detections constitute the dominant residual error. Several categories, including Brown spot, Leaf blast, Leaf smut, and Narrow brown spot, still show relatively high proportions of samples assigned to background, although these values are reduced after introducing AGC-YOLO. Collectively, these results suggest that the combination of the three modules tends to improve inter-class discrimination across datasets that differ in scale and class granularity, indicating a reasonable degree of cross-dataset robustness within the limited set of datasets examined here.

Furthermore, each module was individually validated on test set images, and the corresponding detection results were systematically compared and analyzed. As illustrated in **Figure 16**, all detection outputs were annotated with predicted class labels and confidence scores. From the baseline to the full AGC-YOLO model, false negatives and false positives progressively decreased while detection accuracy steadily increased, thereby fully validating the superior detection performance of AGC-YOLO on the disease detection task.

**Figure 16:**
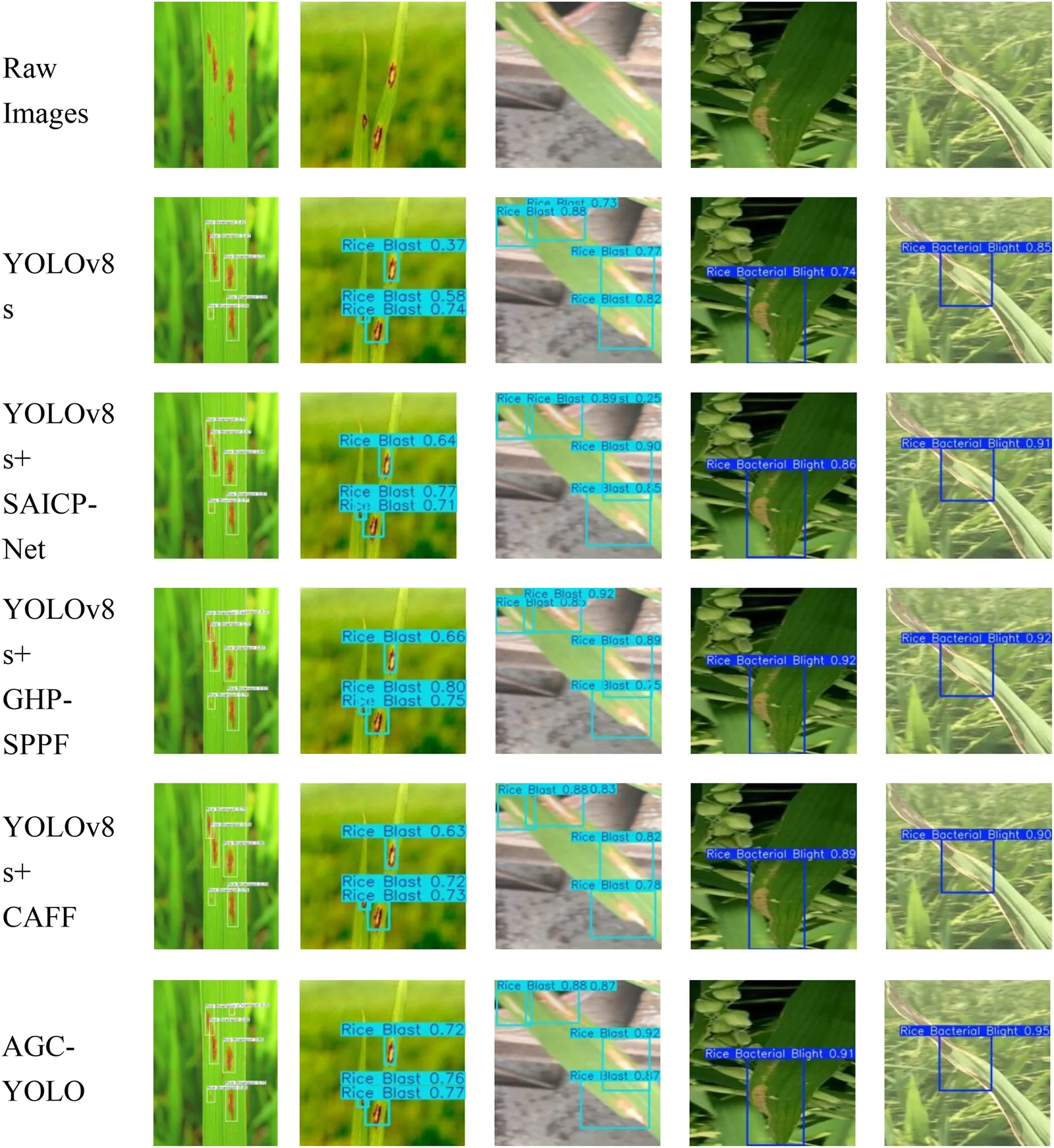
Qualitative comparison of ablation study on rice disease detection. From top to bottom: original image, baseline YOLOv8s, YOLOv8s with SAICP Net, YOLOv8s with GHP-SPPF, YOLOv8s with CAFF, and the complete AGC-YOLO.

Finally, the spatial prior generated by LeafSegNet is qualitatively examined in **Figure 17**. Higher responses are mainly distributed around lesion-associated plant regions, whereas homogeneous background areas generally exhibit lower responses. This indicates that the weakly supervised probability map can provide useful spatial guidance for SAICP Net. The generated response is used only as a soft placement prior rather than as a pixel-accurate segmentation result.

**Figure 17:**
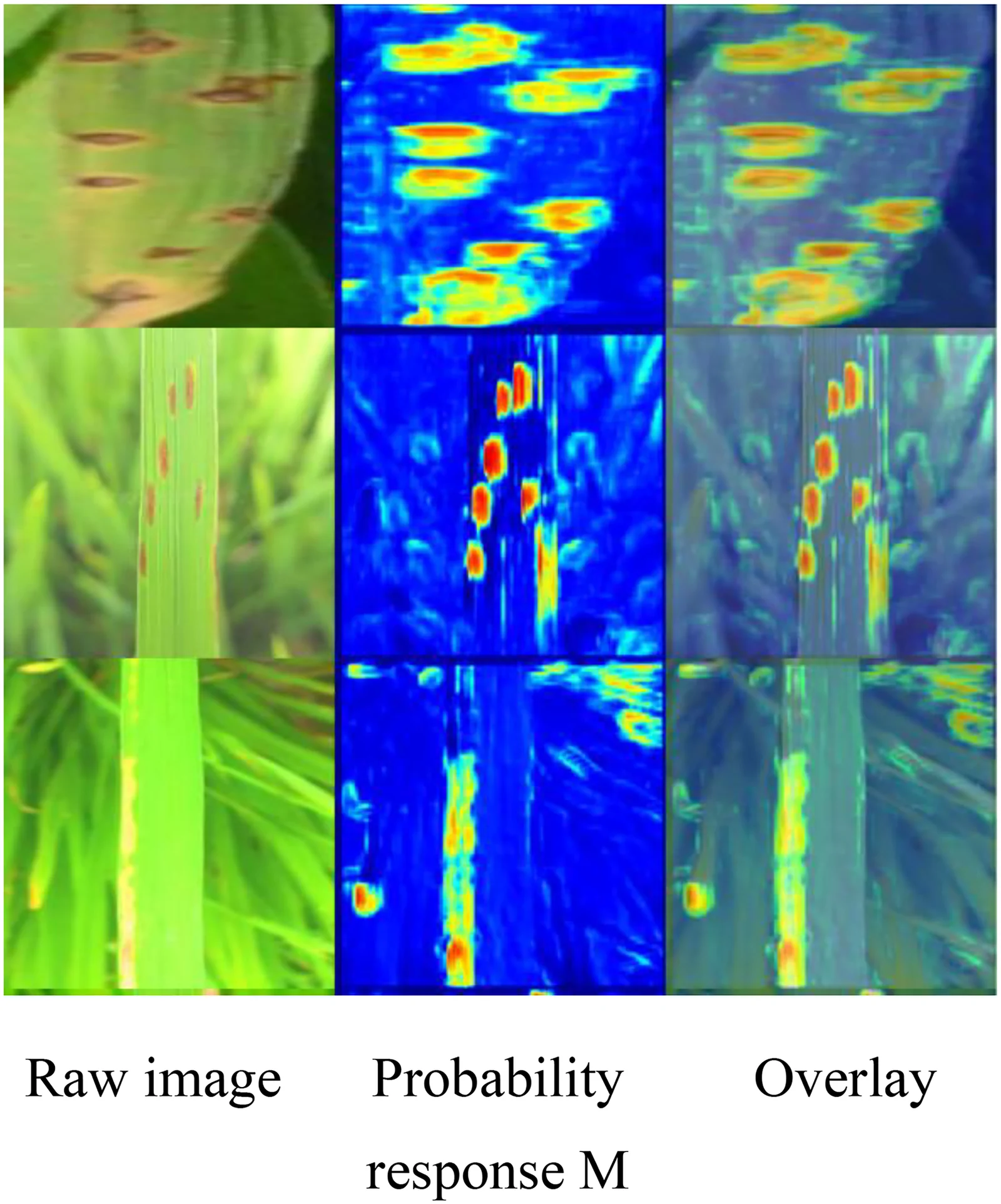
Qualitative visualization of the LeafSegNet soft foreground prior on held-out images. From left to right: original image, probability response M, and overlay.

## 4 Conclusion

To address class imbalance, multi-scale feature information loss, and limited cross-scale feature fusion, this study proposes AGC-YOLO by integrating SAICP Net, GHP-SPPF, and CAFF. Across five random seeds, AGC-YOLO achieved an mAP@0.5 of 93.5 ± 0.6%, representing a 3.0 percentage-point improvement over the YOLOv8s baseline. Additional experiments on the Tomato Leaf Diseases and 11-class Rice Leaf datasets provide supplementary evidence of the transferability of the proposed method, while broader generalization across crop species remains to be investigated.

Several limitations remain. First, the primary dataset is relatively small, particularly the 134-image test set. Second, LeafSegNet provides a weak box-supervised foreground prior rather than pixel-accurate leaf segmentation. Third, all experiments were conducted offline on a high-performance GPU server, and device-level latency, power consumption, and real-world field deployment were not evaluated. Finally, crop-level validation is currently limited mainly to rice and tomato. Future work will therefore investigate dedicated leaf-mask supervision, larger multi-crop datasets, lightweight deployment on edge devices, and prospective validation in real agricultural environments.

## Data Availability

The raw data supporting the conclusions of this article will be made available by the authors, without undue reservation.
